# Structural neuroimaging biomarkers for obsessive-compulsive disorder in the ENIGMA-OCD consortium: medication matters

**DOI:** 10.1038/s41398-020-01013-y

**Published:** 2020-10-08

**Authors:** Willem B. Bruin, Luke Taylor, Rajat M. Thomas, Jonathan P. Shock, Paul Zhutovsky, Yoshinari Abe, Pino Alonso, Stephanie H. Ameis, Alan Anticevic, Paul D. Arnold, Francesca Assogna, Francesco Benedetti, Jan C. Beucke, Premika S. W. Boedhoe, Irene Bollettini, Anushree Bose, Silvia Brem, Brian P. Brennan, Jan K. Buitelaar, Rosa Calvo, Yuqi Cheng, Kang Ik K. Cho, Sara Dallaspezia, Damiaan Denys, Benjamin A. Ely, Jamie D. Feusner, Kate D. Fitzgerald, Jean-Paul Fouche, Egill A. Fridgeirsson, Patricia Gruner, Deniz A. Gürsel, Tobias U. Hauser, Yoshiyuki Hirano, Marcelo Q. Hoexter, Hao Hu, Chaim Huyser, Iliyan Ivanov, Anthony James, Fern Jaspers-Fayer, Norbert Kathmann, Christian Kaufmann, Kathrin Koch, Masaru Kuno, Gerd Kvale, Jun Soo Kwon, Yanni Liu, Christine Lochner, Luisa Lázaro, Paulo Marques, Rachel Marsh, Ignacio Martínez-Zalacaín, David Mataix-Cols, José M. Menchón, Luciano Minuzzi, Pedro S. Moreira, Astrid Morer, Pedro Morgado, Akiko Nakagawa, Takashi Nakamae, Tomohiro Nakao, Janardhanan C. Narayanaswamy, Erika L. Nurmi, Joseph O’Neill, Jose C. Pariente, Chris Perriello, John Piacentini, Fabrizio Piras, Federica Piras, Y. C. Janardhan Reddy, Oana G. Rus-Oswald, Yuki Sakai, João R. Sato, Lianne Schmaal, Eiji Shimizu, H. Blair Simpson, Noam Soreni, Carles Soriano-Mas, Gianfranco Spalletta, Emily R. Stern, Michael C. Stevens, S. Evelyn Stewart, Philip R. Szeszko, David F. Tolin, Ganesan Venkatasubramanian, Zhen Wang, Je-Yeon Yun, Daan van Rooij, ENIGMA-OCD Working Group, Nerisa Banaj, Nuria Bargalló, Marcelo C. Batistuzzo, Daniel Brandeis, Geraldo F. Busatto, Anna Calvo, Valentina Ciullo, Renate Drechsler, Madalena Esteves, Andrea Falini, Yu Fang, Martijn Figee, Martine Fontaine, Margot Gueguen, Sayo Hamatani, Gregory L. Hanna, Bjarne Hansen, Keisuke Ikari, Luisa Lázaro, Ricardo Magalhães, Yasutaka Masuda, Koji Matsumoto, Euripedes C. Miguel, Astrid Morer, Christopher Pittenger, Sara Poletti, Yuki Sakai, Nuno Sousa, Jumpei Takahashi, Anders L. Thorsen, Aki Tsuchiyagaito, Daniela Vecchio, Dick J. Veltman, Susanne Walitza, Anri Watanabe, Xiufeng Xu, Jian Xu, Kei Yamada, Tokiko Yoshida, Mojtaba Zarei, Qing Zhao, Cong Zhou, Froukje E. de Vries, Stella J. de Wit, Daan van Rooij, Guido A. van Wingen, Odile A. van den Heuvel, Ysbrand D. van der Werf, Paul M. Thompson, Odile A. van den Heuvel, Dan J. Stein, Guido A. van Wingen

**Affiliations:** 1 Amsterdam UMC, University of Amsterdam, Department of Psychiatry, Amsterdam Neuroscience, Amsterdam, Netherlands; 2 Department of Physiology, Anatomy and Genetics, Oxford, UK; 3 Department of mathematics and applied mathematics, University of Cape Town, Cape Town, South Africa; 4 Department of Psychiatry, Graduate School of Medical Science, Kyoto Prefectural University of Medicine, Kyoto, Japan; 5 Department of Psychiatry, Bellvitge University Hospital, Bellvitge Biomedical Research Institute-IDIBELL, L’Hospitalet de Llobregat, Barcelona, Spain; 6 Centro de Investigación Biomèdica en Red de Salud Mental-CIBERSAM, Barcelona, Spain; 7 Department of Clinical Sciences, University of Barcelona, Barcelona, Spain; 8 The Margaret and Wallace McCain Centre for Child, Youth and Family Mental Health, Campbell Family Mental Health Research Institute, The Centre for Addiction and Mental Health, Department of Psychiatry, Faculty of Medicine, University of Toronto, Toronto, Canada; 9 Centre for Brain and Mental Health, The Hospital for Sick Children, Toronto, Canada; 10 Department of Psychiatry, Yale University School of Medicine, New Haven, CT 06510 USA; 11 Mathison Centre for Mental Health Research and Education, Hotchkiss Brain Institute, Cumming School of Medicine, University of Calgary, Calgary, Alberta Canada; 12 Department of Psychiatry, Cumming School of Medicine, University of Calgary, Calgary, AB T2N 1N4 Canada; 13 Laboratory of Neuropsychiatry, Department of Clinical and Behavioral Neurology, IRCCS Santa Lucia Foundation, Rome, Italy; 14 Psychiatry and Clinical Psychobiology, Division of Neuroscience, Scientific Institute Ospedale San Raffaele, Milano, Italy; 15 Department of Psychology, Humboldt-Universität zu Berlin, Berlin, Germany; 16 Department of Clinical Neuroscience, Centre for Psychiatric Research and Education, Karolinska Institutet, Stockholm, Sweden; 17 Amsterdam UMC, Vrije Universteit Amsterdam, Department of Psychiatry, Amsterdam Neuroscience, Amsterdam, The Netherlands; 18 Amsterdam UMC, Vrije Universiteit Amsterdam, Department of Anatomy and Neurosciences, Amsterdam Neuroscience, Amsterdam, The Netherlands; 19 Obsessive-Compulsive Disorder (OCD) Clinic Department of Psychiatry National Institute of Mental Health and Neurosciences, Bangalore, India; 20 Department of Child and Adolescent Psychiatry and Psychotherapy, Psychiatric Hospital, University of Zurich, Zurich, Switzerland; 21 University of Zurich and ETH Zurich, Neuroscience Center Zurich, Zurich, Switzerland; 22 McLean Hospital, Harvard Medical School, Belmont, MA 02115 USA; 23 Donders Institute for Brain, Cognition and Behaviour, Radboud University, Nijmegen, Netherlands; 24 Karakter Child and Adolescent Psychiatry University Center, Nijmegen, The Netherlands; 25 Department of Child and Adolescent Psychiatry and Psychology, Institute of Neurosciences, Hospital Clínic Universitari, Barcelona, Spain; 26 Department of Medicine, University of Barcelona, Barcelona, Spain; 27 Centro de Investigación Biomédica en red de Salud Mental (CIBERSAM), Barcelona, Spain; 28 Department of Psychiatry, First Affiliated Hospital of Kunming Medical University, Kunming, China; 29 Institute of Human Behavioral Medicine, SNU-MRC, Seoul, Republic of Korea; 30 Netherlands Institute for Neuroscience, Royal Netherlands Academy of Arts and Sciences, Amsterdam, The Netherlands; 31 Department of Psychiatry, Icahn School of Medicine at Mount Sinai, New York, NY 10029 USA; 32 Department of Psychiatry and Biobehavioral Sciences, University of California, Los Angeles, CA 94612 USA; 33 Department of Psychiatry, University of Michigan, Ann Arbor, MI 48109 USA; 34 Department of Psychiatry and Mental Health, University of Cape Town, Cape Town, South Africa; 35 Department of Neuroradiology, Klinikum rechts der Isar, Technische Universität München, München, Germany; 36 TUM-Neuroimaging Center (TUM-NIC) of Klinikum rechts der Isar, Technische Universität München, München, Germany; 37 Max Planck UCL Centre for Computational Psychiatry and Ageing Research, London, UK; 38 Wellcome Centre for Human Neuroimaging, University College London, London, UK; 39 Research Center for Child Mental Development, Chiba University, Chiba, Japan; 40 Departamento e Instituto de Psiquiatria do Hospital das Clinicas, IPQ HCFMUSP, Faculdade de Medicina, Universidade de Sao Paulo, Sao Paulo, SP Brasil; 41 Shanghai Mental Health Center Shanghai Jiao Tong University School of Medicine, Shanghai, China; 42 De Bascule, Academic Center for Child and Adolescent Psychiatry, Amsterdam, The Netherlands; 43 Department of child and adolescent psychiatry Amsterdam UMC, Amsterdam, The Netherlands; 44 Division of Child and Adolescent Psychiatry, Department of Psychiatry, Icahn School of Medicine at Mount Sinai, New York, NY 10029 USA; 45 Department of Psychiatry, , Oxford University, Oxford, UK; 46 University of British Columbia, Vancouver, BC V6T 1Z4 Canada; 47 Bergen Center for Brain Plasticity, Haukeland University Hospital, Bergen, Norway; 48 Department of Clinical Psychology, University of Bergen, Bergen, Norway; 49 Department of Psychiatry, Seoul National University College of Medicine, Seoul, Republic of Korea; 50 Department of Brain and Cognitive Sciences, Seoul National University College of Natural Sciences, Seoul, Korea; 51 SAMRC Unit on Risk and Resilience in Mental Disorders, Department of Psychiatry, Stellenbosch University, Stellenbosch, South Africa; 52 Institut d’Investigacions Biomèdiques August Pi i Sunyer (IDIBAPS), Barcelona, Spain; 53 Life and Health Sciences Research Institute (ICVS), School of Medicine, University of Minho, Braga, Portugal; 54 ICVS/3B’s, PT Government Associate Laboratory, Braga/Guimarães, Portugal; 55 Clinical Academic Center-Braga, Braga, Portugal; 56 Columbia University Irving Medical Center, Columbia University, New York, NY 10027 USA; 57 The Division of Child and Adolescent Psychiatry, New York State Psychiatric Institute, Columbia University, New York, NY 10027 USA; 58 Department of Clinical Neuroscience, Centre for Psychiatry Research, Karolinska Institutet, Stockholm, Sweden; 59 Department of Psychiatry and Behavioural Neurosciences, McMaster University, Hamilton, ON L8S 4L8 Canada; 60 Department of Neuropsychiatry, Graduate School of Medical Sciences, Kyushu University, Fukuoka, Japan; 61 Division of Child and Adolescent Psychiatry, Jane and Terry Semel Institute For Neurosciences, University of California, Los Angeles, CA 94612 USA; 62 Magnetic Resonance Image Core Facility, IDIBAPS (Institut d’Investigacions Biomèdiques August Pi i Sunyer), Barcelona, Spain; 63 University of Illinois at Urbana-Champaign, Champaign, IL 61820 USA; 64 University of Zürich, University Hospital Zürich, Dept. Neuroradiology, Zürich, Switzerland; 65 University Department of Geriatric Medicine Felix Platter, Basel, Switzerland; 66 ATR Brain Information Communication Research Laboratory Group, Kyoto, Japan; 67 Center of Mathematics, Computing and Cognition, Universidade Federal do ABC, Santo Andre, Brazil; 68 Orygen, Parkville, VIC Australia; 69 Centre for Youth Mental Health, The University of Melbourne, Melbourne, VIC 3052 Australia; 70 Department of Cognitive Behavioral Physiology, Graduate School of Medicine, Chiba University, Chiba, Japan; 71 Center for OCD and Related Disorders, New York State Psychiatric Institute, New York, NY 10032 USA; 72 Pediatric OCD Consultation service, Anxiety Treatment and Research Center, St. Joseph’s HealthCare, Hamilton, ON L9C 0E3 Canada; 73 Offord Child Center, McMaster University, Hamilton, ON L8S 4L8 Canada; 74 Department of Psychobiology and Methodology of Health Sciences, Universitat Autònoma de Barcelona, Barcelona, Spain; 75 Beth K. and Stuart C. Yudofsky Division of Neuropsychiatry, Department of Psychiatry and Behavioral Sciences, Baylor College of Medicine, Houston, TX 77030 USA; 76 Department of Psychiatry, New York University Langone School of Medicine, New York, NY 10016 USA; 77 Nathan Kline Institute for Psychiatric Research, Orangeburg, NY 10962 USA; 78 Olin Neuropsychiatry Research Center, Hartford Hospital, Hartford, CT 06106 USA; 79 Department of Psychiatry, Yale University School of Medicine, New Haven, 06510 USA; 80 British Columbia Children’s Hospital, Vancouver, BC V6H 3N1 Canada; 81 British Columbia Mental Health and Addictions Research Institute, Vancouver, BC V6H 3N1 Canada; 82 Icahn School of Medicine at Mount Sinai, New York, NY 10029 USA; 83 James J. Peters VA Medical Center, Bronx, New York, NY 10468 USA; 84 Institute of Living/Hartford Hospital, Hartford, CT 06119 USA; 85 Yale University School of Medicine, New Haven, CT 06510 USA; 86 Shanghai Key Laboratory of Psychotic Disorders, Shanghai, China; 87 Seoul National University Hospital, Seoul, Republic of Korea; 88 Yeongeon Student Support Center, Seoul National University College of Medicine, Seoul, Republic of Korea; 89 Department of Cognitive Neuroscience, Donders Institute for Brain, Cognition and Behaviour, Donders Centre for Cognitive Neuroimaging, Radboud University Medical Centre, Nijmegen, The Netherlands; 90 Imaging Genetics Center, Stevens Institute for Neuroimaging and Informatics, Keck School of Medicine, University of Southern California, Los Angeles, CA 90007 USA; 91 SAMRC Unit on Risk and Resilience in Mental Disorders, Department of Psychiatry and Neuroscience Institute, University of Cape Town, Cape Town, South Africa; 92 Image Diagnostic Center, Hospital Clínic, Barcelona, Spain; 93 Department of Child and Adolescent Psychiatry and Psychotherapy, Psychiatric Hospital, University of Zurich, Zurich, Switzerland; 94 Department of Child and Adolescent Psychiatry and Psychotherapy, Central Institute of Mental Health, Mannheim, Medical Faculty Mannheim/Heidelberg University, Mannheim, Germany; 95 Neuroscience Center Zurich, University of Zurich and ETH Zurich, Zurich, Switzerland; 96 Laboratory of Psychiatric Neuroimaging (LIM21), Hospital das Clinicas HCFMUSP, Faculdade de Medicina, Universidade de Sao Paulo, Sao Paulo, Brazil; 97 Department and Institute of Psychiatry, Faculty of Medicine, University of Sao Paulo, Sao Paulo, Brazil; 98 Center for Interdisciplinary Research on Applied Neurosciences (NAPNA), University of Sao Paulo, Sao Paulo, Brazil; 99 Department of Neurosciences, Psychology, Drug Research and Child Health (NEUROFARBA), University of Florence, Firenze, Italy; 100 Department of Child and Adolescent Psychiatry and Psychotherapy, Psychiatric Hospital, University of Zurich, Zurich, Switzerland; 101 Neuroradiology, Division of Neuroscience, Scientific Institute Ospedale San Raffaele, Milano, Italy; 102 Department of Psychiatry, University of Michigan, Ann Arbor, MI 48109 USA; 103 Department of Psychiatry, Icahn School of Medicine at Mount Sinai, New York, 10029 USA; 104 Department of Anatomy and Neurosciences, Amsterdam UMC, Vrije Universiteit Amsterdam, Amsterdam Neuroscience, Amsterdam, The Netherlands; 105 Department of Radiology, Chiba University Hospital, Chiba, Japan; 106 Department of Psychiatry, Yale University School of Medicine, New Haven, CT 06510 USA; 107 Amsterdam UMC, Vrije Universteit Amsterdam, Department of Anatomy and Neurosciences, Amsterdam, The Netherlands; 108 Laureate Institute for Brain Research, Tulsa, OK 74136 USA; 109 Department of Internal Medicine, First Affiliated Hospital of Kunming Medical University, Kunming, China; 110 Department of Radiology, Graduate School of Medical Science Kyoto Prefectural University of Medicine, Kyoto, Japan; 111 Institute of Medical Science and Technology, Shahid Beheshti University, Tehran, Iran

**Keywords:** Neuroscience, Diagnostic markers, Neuroscience, Diagnostic markers

## Abstract

No diagnostic biomarkers are available for obsessive-compulsive disorder (OCD). Here, we aimed to identify magnetic resonance imaging (MRI) biomarkers for OCD, using 46 data sets with 2304 OCD patients and 2068 healthy controls from the ENIGMA consortium. We performed machine learning analysis of regional measures of cortical thickness, surface area and subcortical volume and tested classification performance using cross-validation. Classification performance for OCD vs. controls using the complete sample with different classifiers and cross-validation strategies was poor. When models were validated on data from other sites, model performance did not exceed chance-level. In contrast, fair classification performance was achieved when patients were grouped according to their medication status. These results indicate that medication use is associated with substantial differences in brain anatomy that are widely distributed, and indicate that clinical heterogeneity contributes to the poor performance of structural MRI as a disease marker.

## Introduction

Obsessive-compulsive disorder (OCD) is a severe and debilitating condition that occurs in 2–3% of the population^1^. It is characterized by recurrent, intrusive, irrational and distressing thoughts (obsessions) and repetitive behaviors or mental acts (compulsions)^2^. So far, no biomarkers that aid differential diagnosis are available, and diagnosis relies entirely on recognition of characteristic symptoms assessed by clinical interview^3^. Many neuroimaging studies have provided evidence for abnormalities in cortico-striato-thalamo-cortical (CSTC) circuits, as well as distributed changes in limbic, parietal and cerebellar regions^4,5^. These findings have recently been confirmed by different meta-analyses and mega-analyses of neuroimaging studies, based on results that were reported in the literature or by using original data within different consortia^6–10^. However, inference has been at the group-level, and the small effect sizes reported preclude clinical application.

Analytic tools such as multivariate pattern analysis (MVPA) enable inference at the individual-level, which may result in better discrimination^3,11^. MVPA techniques can be used to develop predictive models that extract common patterns from neuroimaging data to classify individuals based on their diagnosis. A major advantage of MVPA compared to traditional methods of analysis is its ability to use inter-regional correlations to detect subtle and spatially distributed effects^4^. Therefore, MVPA seems particularly well suited for neuroimaging analyses in OCD, as abnormalities are typically distributed across the brain^12,13^. Previous MVPA studies have been able to distinguish OCD patients from controls with accuracies ranging from 66–100%^14^. Although these results are promising, sample sizes have typically been small, limiting model performance optimization and leading to high variance in estimated accuracy, which may result in reporting optimistic or pessimistic classification rates^15^. In addition, most studies have been performed using data from one research center to minimize technical (e.g., scanner hardware, protocols, and diagnostic assessment) and clinical (e.g., age, medication status, disease chronicity, and severity) heterogeneity. It is therefore not clear whether the MVPA results obtained from these monocenter studies generalize well to other centers, which would be required for clinical application^16–18^. Interestingly, whereas classification accuracies of monocenter studies only tend to increase with larger samples^19,20^, accuracies for multicenter studies in other psychiatric disorders such as schizophrenia and autism tend to be lower with increasing sample size^12,15–17^. This paradoxical effect of lower classification accuracy with larger samples has been attributed to larger sample heterogeneity^21^, which inevitably increases when combining data from different centers. Here, we used data from the Enhancing Neuro-Imaging and Genetics through Meta-Analysis (ENIGMA) OCD consortium, including 4372 participants recruited at 36 research institutes around the world, with a full range of technical and clinical heterogeneity. We assessed the ability of MVPA to distinguish OCD patients from healthy controls using structural neuroimaging data at the individual subject level. We investigated machine learning classification performance in both single-site and multi-site samples using different validation strategies to assess generalizability. Furthermore, the large sample size enables investigation of the influence of clinical heterogeneity by stratification and subsampling, in order to assess the influence of clinical variability on classification accuracy.

## Materials and methods

### Study population

The ENIGMA-OCD working group includes 46 data sets from 36 international research institutes, with neuroimaging and clinical data from adult (≥18 years) and pediatric (<18 years) samples. In total, we analyzed data from 4372 participants, including 2304 OCD patients (*n* = 1801 adult, *n* = 503 child) and 2068 healthy controls (HC; i.e., free of psychopathology; *n* = 1629 adult, *n* = 439 child), with 38 of 46 datasets identical to those described in previous mega-analyses by this working group^6,7,22^. All participating sites obtained permission from their local institutional review boards or ethics committees to provide anonymized data for analysis, and all study participants provided written informed consent. Demographic and clinical characteristics of each site are detailed in supplementary Table S1. A complete overview of instruments used to obtain diagnosis and clinical information can be found elsewhere (Data Supplement 1, Supplementary Section S1)^7^. Diagnosis was determined in accordance with DSM^2^; MINI and SCID were used for adult samples and K-SADS, MINI-KIDS and ADIS were used for pediatric samples^23–27^.

### MRI processing

Structural T1-weighted brain MRI scans were acquired and processed locally at each site. Image acquisition parameters are listed elsewhere^7^. Parcellations were performed using FreeSurfer software version 5.3 (http://surfer.nmr.mgh.harvard.edu), following standardized ENIGMA protocols to harmonize analyses and quality control procedures across multiple sites (see http://enigma.usc.edu/protocols/imaging-protocols/). Mean values of parcellations of 34 cortical (Desikan-Killiany atlas-based^28^) and 7 subcortical gray matter structures per hemisphere, lateral ventricle volumes, two whole-hemisphere measures and total intracranial volume were extracted, visually inspected and statistically evaluated for outliers (quality assurance is reported elsewhere^7^). Brain regions (features) used for classification included cortical thickness (CT), surface area (SA) and subcortical volumes of ROIs, two lateral ventricular and intra-cranial volumes (ICV), and two whole-hemisphere measures for SA and CT.

### Multivariate classification and validation

Participants with >10% missing entries were excluded (*n* = 276), and median imputation was used for missing MRI data on the training set. Continuous features were centered around median zero and scaled according to their interquartile range. FreeSurfer variables were combined with covariates age, sex, and site by concatenating individual feature vectors. Categorical covariates were one-hot encoded prior to classification. All analyses were performed separately for pediatric and adult patients, and both groups combined. Common MVPA classifiers were applied: support vector machine (SVM) with linear and non-linear (radial-basis-function (RBF)) kernels, logistic regression (LR) with L1 and L2 regularization, Gaussian processes classification (GPC) with a linear kernel, and two decision-tree based ensemble methods, namely the random forest classifier (RFC) and the XGBoost (XGB) algorithm^29–32^. A neural network was also implemented (fully connected; 3 hidden layers with 60, 40, and 20 nodes respectively). SVM and LR classifiers were combined with and without automatic dimensionality reduction via principal component analysis (PCA), using the minimal number of components explaining 90% of the variance. Hyper-parameters for SVM (linear and non-linear), LR and XGB were optimized using nested cross-validation; RFC and GPC were tuned following recommendations. Details on handling missing data, model implementation and hyper-parametrization can be found in Supplementary Methods (and supplementary Fig. S1 for assessment of different imputation strategies). The primary performance metric was the area under the receiver operator curve (AUC) and reported metrics are averaged across CV folds^33^. Balanced accuracy, sensitivity and specificity are reported in the supplement.

Multi-site classification of OCD patients versus HC was assessed using different cross-validation (CV) approaches. First, we assessed multi-site classification using 10-fold site-stratified CV to obtain maximally homogeneous train-test splits, with approximately the same number of subjects in each fold and the same proportion of samples coming from each site (also referred to as ‘internal validation’). Next, we addressed leave-one-site-out (LOSO) CV, in which all but one site were used to train the models while the left out site was used to assess model performance (external validation). This procedure is then repeated so that each site is used once as a test set. LOSO-CV may result in large between-sample heterogeneity of training and test sets, resulting in lower classification performance^34^. Because LOSO-CV has different fold sizes, we additionally performed site-stratified CV with varying fold sizes, in which the number of CV folds and respective test-fold sizes are set to match those of LOSO-CV. This was done to evaluate whether differences between site-stratified and LOSO-CV performance were due to differences in heterogeneity or test-fold size variance. Finally, we also performed single-site predictions using repeated 5-fold CV (with 10 repeats) to assess classification performance for individual sites with reduced heterogeneity. For a schematic representation of LOSO and site-stratified CV see supplementary Fig. S2. Statistical significance of model performance and 95% confidence intervals were assessed through the obtained AUC scores using the Mann–Whitney-U statistic for non-parametric testing (see Supplement for details)^33,35,36^. The Bonferroni-corrected level of significance was set at alpha = 0.05 for the number of classifiers and comparisons (3 CV types × 10 classifiers for multi-site classifications; alpha = 0.05/30). As previous work from ENIGMA-OCD has identified distinct alterations in pediatric and adult patients, we performed all multi-site classification analyses for pediatric (<=18 years of age) and adult (>18 years of age) data separately, as well as both age groups combined^6,7^.

### Clinical variables and sensitivity analysis

To explore the effects of clinical heterogeneity on classification performance, we selected subgroups with particular demographic and clinical characteristics: medication use, OCD severity, age of onset (AO) and duration of illness. Classifications performed were HC vs. low (YBOCS <= 24; mild-moderate^37^) and high severity (YBOCS > 24; moderate-severe) OCD; HC vs. early (<18 yrs) and late AO (>= 18 yrs) OCD; HC vs. short (<=7 yrs) and long duration (>7 yrs) OCD; and HC vs. unmedicated and medicated OCD. For disease duration and severity, median splits were used to define groups; the 18 year threshold for AO was chosen in line with prior ENIGMA-OCD mega-analyses^6,7^. Finally, as particular clinical variables can co-occur, we performed a post-hoc sensitivity analysis to investigate the effects of potential clinical covariance for results with AUC ≥ 0.8. First, correlations between all clinical features were computed using point-biserial correlations between dichotomous and continuous variables, phi correlation for dichotomous variables and Pearson correlation for continuous variables. Only those clinical features that were significantly correlated (Bonferroni-corrected) were investigated further by rerunning previously described classifications, but now using samples further split according to their correlated features (e.g., HC vs. unmedicated, short duration OCD patients; etc.). The use of more homogenous subsamples is expected to improve classification performance, while reducing sample size itself is expected to decrease performance. If classifications are relatively unaffected by further splitting, the correlated clinical variable did not have a large influence on the classification results.

### Feature importance

To assess which brain regions and clinical variables contributed most to classification we used feature importance extracted from RFC combined with a permutation testing framework (see Supplementary Methods)^38^.

## Results

### Multi-site classification

**Fig. 1:**
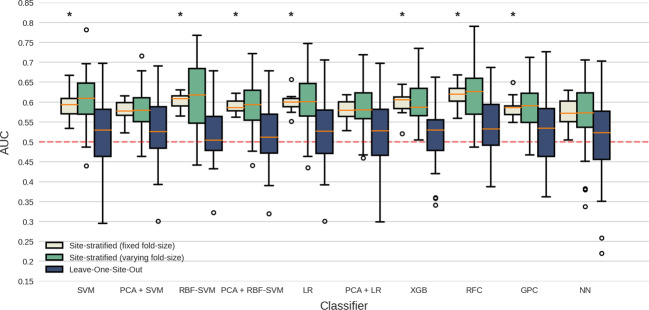
Performance for multi-site classification using different algorithms and cross-validation schemes. Boxplots summarize AUC scores obtained across CV-folds; dashed line represents chance-level performance and asterisks indicate scores significantly different from chance (Mann–Whitney-U statistic; *p* < 0.05 Bonferroni corrected (10 classifiers × 3 CV types), see Supplement for details). *SVM* Support Vector Machine, *PCA* Principal Component Analysis, *RBF* Radial Basis Function, *LR* Logistic Regression, *GPC* Gaussian Processes Classification, *RFC* Random Forest Classifier, *XGB* XGBoost, *NN* Neural Network.

Three different CV approaches were used to assess the influence of sample heterogeneity. Results using various classification algorithms are summarized in Fig. 1. Classification performance (AUC) using site-stratified CV (with training on combined samples and equal fold sizes) ranged between 0.57 (95% confidence intervals (CI) = 0.51–0.63; p_corrected_ = 0.19) and 0.62 (95% CI = 0.56–0.67; p_corrected_ < 0.001) across different classifiers. All models had statistically significant performance after multiple comparison corrections except for PCA + LR, PCA + SVM and NN classifiers. LOSO-CV led to lower classification performance; 0.51 (95% CI = 0.4–0.62; p_corrected_ = 1) to 0.54 (95% CI = 0.42–0.65; p_corrected_ = 1) AUC with relatively high variance across folds (SD = 0.07–0.11) and no classifiers surviving multiple comparison corrections. AUC values obtained through site-stratified CV with varying fold sizes were similar to site-stratified CV results with equal fold sizes, ranging between 0.56 (95% CI = 0.45–0.67; p_corrected_ > 0.99) and 0.62 (95% CI = (0.51–0.73); p_corrected_ = 0.55). However, variance across CV-folds was higher and comparable to that from LOSO-CV (SD; site-stratified fixed: 0.02–0.04; site-stratified variable: 0.05–0.08; LOSO: 0.07–0.11). A complete overview of classification results is provided in supplementary Table S2. Multi-site classification with site-stratified CV (with equal fold sizes), performed separately on pediatric and adult samples yielded similar results, ranging from 0.55 (95% CI = 0.43–0.67; p_corrected_ = 1) to 0.62 (95% CI = 0.51–0.74; p_corrected_ = 0.71) and 0.56 (95% CI = 0.5–0.62; p_corrected_ = 0.69) to 0.61 (95% CI = 0.55–0.67; p_corrected_ = 0.008) AUC, respectively (see supplementary Tables S3-4). As site-stratified CV with equal fold-sizes resulted in the best performances, we used this strategy for further evaluation of intra-site performance and the influence of clinical variables. RFC classification performance is reported here by default, as differences between classifiers were minimal and this model was also used to extract feature importance.

### Single-site classification

**Fig. 2:**
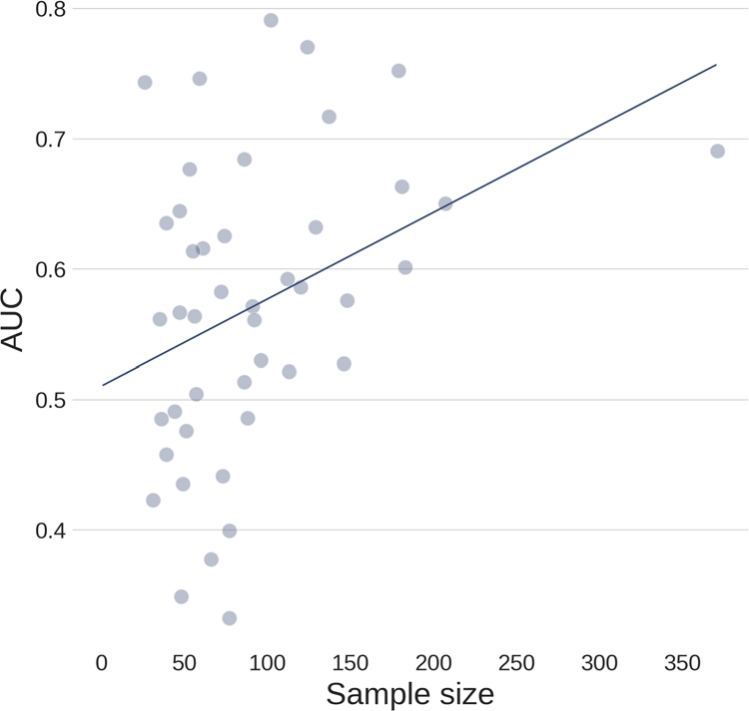
Scatterplot illustrating relationship between number of participants and classification performance across sites. Only RFC classifier performance averaged across CV-folds and repeats are plotted (Spearman correlation; *r*_S_ = 0.37, *p* = 0.014).

Single-site classification performance with 10-fold CV varied greatly, with AUCs ranging between 0.30–0.89 across different sites and classifiers (see supplementary Table S5). Figure 2 summarizes RFC performances for each individual site. We assessed the correlation between the number of participants in each site and its obtained classification performance (AUC averaged over CV folds), which was significant (*r*_S _= 0.37, *p* = 0.014). In addition, we investigated the relationship between single-site classification performance and the following clinical variables of interest: mean and standard deviation of AO, duration, severity and the proportion of medicated patients and its standard deviation. None of these clinical variables showed a significant correlation with classification performance.

### Clinical variables and sensitivity analysis

**Fig. 3:**
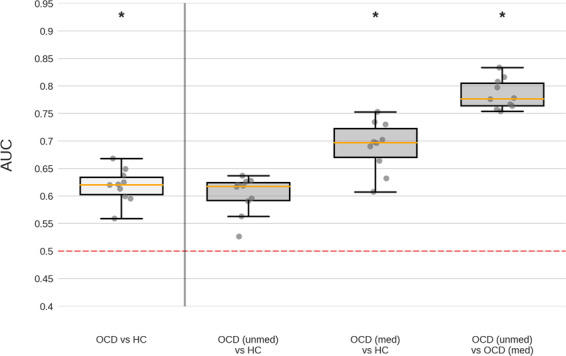
Performance for classification between subgroups of patients based on medication status. Only RFC classifier performance for combined (pediatric and adult) data is shown here; Boxplots summarize AUC scores obtained across CV-folds; dashed line represents chance-level performance and asterisks indicate scores significantly different from chance (Mann–Whitney-U statistic; *p* < 0.05 Bonferroni corrected (10 classifiers × 3 CV types), see Supplement for details). *unmed* unmedicated, *med* medicated.

To assess the influence of different clinical variables on classification performance, we repeated the analysis for specific subgroups split according to medication use, AO, disease duration, and severity. A complete overview is provided in supplementary Tables S6(a–d), and results using RFC on combined data with age, sex and site as covariates are reported below. Medicated OCD vs. HC classification resulted in 0.69 AUC (95% CI = 0.63–0.75; p_corrected_ < 0.001), unmedicated OCD vs. HC in 0.60 (95% CI = 0.54–0.67; p_corrected_ = 0.03), and medicated vs. unmedicated OCD in 0.78 (95% CI = 0.72–0.85; p_corrected_ < 0.001) (see Fig. 3). XGB performance was notably higher for medicated vs. unmedicated OCD classification with an AUC of 0.86 (95% CI = 0.78–0.9; p_corrected_ < 0.001). Early AO OCD vs. HC classification resulted in 0.68 AUC (95% CI = 0.62–0.75; p_corrected_ < 0.001), late AO OCD vs. HC in 0.73 (95% CI = 0.67–0.79; p_corrected_ < 0.001), and early vs. late AO in 0.81 (95% CI = 0.74–0.88; p_corrected_ < 0.001). As no late AO patients were present in pediatric samples, classifications were re-run on adult samples only, resulting in 0.65 AUC (95% CI = 0.57–0.72;p_corrected_ = 0.01) for early AO vs. HC, 0.70 (95% CI = 0.63–0.76; p_corrected_ < 0.001) for late AO vs. HC, and 0.73 (95% CI = 0.64–0.82;p_corrected_ < 0.001) for early vs. late AO. Classification of short disease duration OCD vs. HC resulted in 0.68 AUC (95% CI = 0.61–0.75;p_corrected_ < 0.001), long disease duration vs. HC in 0.71 (95% CI = 0.65–0.78; p_corrected_ < 0.001), and short vs. long duration in 0.78 (95% CI = 0.7–0.85; p_corrected_ < 0.001). Finally, low severity OCD vs. HC classification resulted in 0.60 AUC (95% CI = 0.53–0.67; p_corrected_ = 0.15), high severity OCD vs. HC in 0.61 (95% CI = 0.54–0.68; p_corrected_ = 0.04), and low vs. high severity OCD in 0.58 (95% CI = 0.49–0.66; p_corrected_ = 1).

Correlation analysis between medication status and other clinical variables only showed a significant association with disease duration (r = −0.094; p_corrected_ < 10^−05^; Bonferroni-corrected). We therefore performed additional medication status classifications after splitting patients for disease duration (e.g., HC vs. medicated + short duration OCD; HC vs. unmedicated + short duration OCD, etc.). Classifications with or without splitting patients for disease duration were comparable (see supplementary Tables S7(a–c) for full overview).

### Influence of covariates

As seen in supplementary Table S1, several sites included only medicated patients whereas other sites only included non-medicated patients. To assess whether the high performance reported above for classifying medication status could be explained by site-differences in the covariates (e.g., site ID) rather than neuroimaging data, we performed the following control experiments. We reran both medication and main diagnosis classifications without using covariates (using only brain data), using covariates only, and by using neuroimaging data after removing the effect of the covariates using multiple linear regression. To maintain independence between the training and test sets, regression coefficients were estimated on the training data and applied to the test data.

Results for the main classifications between OCD patients and HC using neuroimaging data only resulted in AUC of 0.61 (95% CI = 0.55–0.66; p_corrected_ = 0.001), while use of covariates only resulted in AUC of 0.58 (95% CI = 0.52–0.64; p_corrected_ = 0.04), and using neuroimaging data following correction for covariates resulted in AUC of 0.58 (95% CI = 0.52–0.64; p_corrected_ = 0.02). These results show that the covariates contain sufficient information to distinguish patients from HC, but the results were otherwise comparable to the AUCs reported above for classifications using the covariates as features.

Classification for medicated patients vs. HC using neuroimaging data only resulted in AUC of 0.66 (95% CI = 0.60–0.73; p_corrected_ < 0.001) and AUC of 0.70 (95% CI = 0.64–0.76; p_corrected_ < 0.001) for SVM-RBF. Using covariates only resulted in AUC of 0.71 (95% CI = 0.65–0.77; p_corrected_ < 0.001), and neuroimaging data following correction for covariates resulted in AUC of 0.59 (95% CI = 0.52–0.66; p_corrected_ = 0.053), and higher AUC of 0.62 (95% CI = 0.55–0.69; p_corrected_ = 0.007) for XGB. Next, classification of unmedicated patients vs. HC using neuroimaging data only resulted in an AUC of 0.58 (95% CI = 0.51–0.68; p_corrected_ = 0.12) with significant classifier performance for PCA + SVM-RBF with AUC of 0.61 (95% CI = 0.55–0.68; p_corrected_ = 0.007). Using covariates only resulted in AUC of 0.64 (95% CI = 0.58–0.73; p_corrected_ < 0.001), and using neuroimaging data following correction for covariates resulted in AUC of 0.52 (95% CI = 0.45–0.59; p_corrected_ = 1). Finally, classification of medicated vs. unmedicated patients using neuroimaging data only resulted in AUC of 0.74 (95% CI = 0.67–0.81; p_corrected_ < 0.001), using covariates only in AUC of 0.84 (95% CI = 0.78–0.92; p_corrected_ < 0.001), and using neuroimaging data after correction for covariates resulted in AUC of 0.59 (95% CI = 0.50–0.67; p_corrected_ = 0.2). A full overview of these control experiment results can be found in supplementary Tables S8–9. These results show that the covariates contained sufficient information to classify these subgroups, and especially to distinguish between medicated vs. unmedicated patients, as subgroups were already partially defined by site. Nevertheless, the classifications using neuroimaging data only were comparable to the classifications reported above when covariates were added as features. However, correcting the neuroimaging data for covariates lowered the performance substantially compared to adding the covariates as features, suggesting that neuroimaging data were partially related to the covariates (e.g., different scanners at different sites).

### Feature importance

We investigated which brain regions (features) contributed most to OCD vs. HC classifications for site-stratified CV only, using the feature importance values from RFC and permutation testing, and as we were interested in brain regions rather than the influence of the covariates, we focused on classifications using brain data only following multiple linear regression of confounding factors. No features were selected consistently (survived false discovery rate (FDR) correction in >50% CV-folds) for the main analyses (OCD patients vs. HC classification) in either pediatric, adult or combined samples. However, for medicated vs. unmedicated OCD classification in combined samples, 24 significant and consistently selected features were found. In addition, 12 features were found for early vs. late AO patients classifications in combined samples. A complete overview of these findings (including features importance for medication classification in adult samples) can be found in supplementary Tables S10–11.

## Discussion

We found that MVPA of parcellated structural neuroimaging data is unable to provide accurate distinction between OCD cases and HC. Classification of the complete sample using site-stratified CV ranged between an AUC of 0.57 and 0.61, which is not sufficient for clinical application. Differences in performance between classifiers were minimal. Similar results were obtained for classifications performed separately on pediatric and adult samples. When validated on completely new data from other sites using LOSO-CV, model performance hardly exceeded chance-level (0.5 AUC).

Our findings highlight the impact of validation schemes on classification performance and suggest poor discrimination between OCD patients and HC when combining data from multiple sites. In contrast, discrimination between subgroups of patients based on medication status enabled fair individual subject classification. However, our control experiments indicated that non-brain covariates such as age, sex and site can heavily affect classification performance, dependent on the relation between the structural neuroimaging data and those covariates. Yet, even after removal of the covariate effects, the results still indicated that medication use is associated with substantial differences in brain anatomy that are widely distributed, whereas gross gray matter anatomy of patients with OCD was comparable to that of healthy controls. At the same time, this also suggests that clinical heterogeneity contributes to the poor performance of structural MRI as a disease biomarker.

Few diagnostic classifiers have been applied to OCD across multiple scanners and sites. Prior studies using structural MRI data to classify OCD using single-site samples yielded accuracies ranging from 0.72 to 0.93^14^. The wide range of performances observed in our individual site classification is in agreement with the published literature. Such a wide range may in part be explained by sample size, as larger samples tended to have higher AUC values^16,19,34^. However, this relationship does not necessarily hold true for large-scale multicenter studies, due to heterogeneity that arises from pooling samples with different scanning parameters, processing pipelines, inclusion criteria, demographic and clinical characteristics^14,21^. All these factors can impact the data and obscure a pattern of abnormalities shared by all patients. Monocenter studies that minimize heterogeneity may therefore yield higher classification performances, but limit the generalizability to new, unseen data and its use in clinical practice^16,17^. Thus, whereas small monocenter studies focus on answering a specific question about their patient population, large multicenter studies assume that a fundamental pattern of the disorder of interest can be detected despite the presence of heterogeneity, and both are geared toward answering complementary questions about a particular disorder^18,39^. Our LOSO-CV results demonstrated that structural MRI features do not provide a biomarker that enables generalization to new sites.

Multi-site classification within subgroups, split according to medication status, resulted in fair performance even after accounting for correlated clinical variables (i.e., disease duration) through additional splits. Evidence from rodent studies suggests that serotonin reuptake inhibitors (SRIs) mediate neuroplasticity in various cortical and subcortical structures through glio-genesis and neuro-genesis^40–42^. However, little is known about how these findings might translate to humans and what the effects of long-term medication use are^43^. The few longitudinal studies with small samples suggest that SRI treatment normalizes brain volumes. One study reported significantly larger thalamic volumes in twenty-one treatment-naïve pediatric patients compared to HC and that these differences decreased following paroxetine treatment^44^. Another study reported that smaller putamen volume in treatment-naïve patients was no longer detectable in the thirteen patients that were treated with fluoxetine^45^. Nonetheless, it remains unclear whether these structural changes are related to medication use or to symptom improvement. In contrast to these normalizing effects of SRIs, the previous univariate meta-analyses and mega-analyses of the ENIGMA-OCD study found significantly thinner cortices in medicated adult OCD patients and smaller cortical surface area in medicated pediatric OCD patients, but could not detect significant differences in cortical and subcortical gray matter between unmedicated OCD patients and HC, with the exception of larger thalamic volumes in unmedicated pediatric OCD patients^6,7^. Together, these cross-sectional studies suggest that medication use alters brain structure rather than necessarily normalizing it to the level of healthy individuals, an hypothesis which needs to be assessed in appropriately powered longitudinal studies.

The identification of which brain regions contributed most to the classification resulted from a multivariate analysis, and the localization of these regions should therefore be interpreted with caution. MVPA techniques typically result in better discriminative ability between groups compared to standard univariate analyses by taking the distributed nature of effects into account, but they do not provide inherent localization information (i.e., attributing effect sizes to individual ROIs) as all features used for prediction are considered as a whole. We derived individual feature importance from the RFC classifier using permutation-based inference to find brain features that contributed both significantly and consistently (across CV folds) to classification performance. Feature importance was derived from classifications using neuroimaging data after regression of covariates to avoid any undesirable effects on the interpretation of weights caused by non-imaging features. No feature importance obtained for the main classifications (OCD patients vs. HC) in either pediatric, adult or combined samples was statistically significant. This is likely due to the low classification performances obtained, suggesting that the features used are either too noisy or non-informative for main diagnosis predictions and unable to achieve statistical significance and consistency across folds. Similarly, no significant features were found for medicated OCD vs. HC and unmedicated OCD vs. HC classifications. On the other hand, significant features were found that enabled multivariate classifications for medicated vs. unmedicated OCD in adult and combined samples. These included widespread cortical thickness in frontal and temporal regions, including the left inferior temporal gyrus, medial orbital frontal and bilateral transverse temporal cortex, left insula, and bilateral anterior cingulate cortex, as well as surface area of the right entorhinal, left paracentral and bilateral temporal cortex, and left thalamus, pallidum, and ventricle volumes. A full overview of these significant features, as well as those found for early vs. late onset OCD classification, can be found in supplementary Tables S10-11.

The brain regions that were detected in the multivariate analysis are partially consistent with the results from previous univariate ENIGMA-OCD meta-analyses and mega-analyses^6,7^. Medicated adult OCD patients showed thinner frontal, temporal and parietal cortices, and smaller hippocampal and larger pallidum volumes compared to HC, whereas no differences were found for unmedicated adult patients. Although we were unable to detect significant features for medicated patients and unmedicated patients vs. HC classifications, these earlier results fit with the finding that classification performance for medicated patients vs. HC was better than for unmedicated patients vs. HC, as differences in brain anatomy of unmedicated patients appear to be minimal. Interestingly, the classification between medicated vs. unmedicated patients was even better, which suggests that this results from the minimization of heterogeneity in stratified patient groups as opposed to the larger heterogeneity seen in case-control comparisons. Finally, as the medication used for treating OCD is also used for treatment of many other psychiatric disorders, we anticipate that these results are not specific to OCD.

Another point that deserves emphasis concerns the different ways of dealing with confounding non-imaging variables (e.g., age and sex) when using neuroimaging data for MVPA classification. Although recent studies were unable to detect differences in predictive performance when comparing different approaches for dealing with confounds in MVPA studies (nor differences in the weights assigned by the models^46,47^), results from our confound control experiments suggest otherwise. We chose to add the covariates age, sex and data collection site ID directly as features to our model as our initial approach. The underlying principle of this approach is that all relevant variables should be included in the model and that their relative contribution to the final predictive model will be recovered during model training, without the need for manual confound adjustment procedures^47^. This approach resulted in high classification performance for medication classifications (>0.8 AUC). However, several sites included only medicated patients while in others no patients had received medication, which could suggest that this high performance was achieved through classifiers detecting site-effects directly from covariates (e.g., site ID) rather than brain data. The latter is supported by the finding that classifications using covariates only (age, sex, and site) also resulted in high AUC (>0.8 AUC), whereas regressing these covariates out from brain data resulted in lower performance, with only classifications for medicated vs. control classification remaining significant (supplementary Tables S9). Our control experiments also show that the FreeSurfer data itself is likely to be confounded by site-effects as well, as classifications using brain data only (without regressing out covariates) resulted in relatively high classification performance for medicated OCD vs. HC and medicated vs. unmedicated OCD classifications (with 0.70 and 0.75 AUC, respectively). This could be explained by classifiers being able to identify sites through specific sample characteristics (demography and inclusions criteria used) resulting in different brain anatomy, and methodical differences such as types of scanner and imaging protocols used. Interestingly, control experiments for main diagnosis classifications showed that these were relatively unaffected by different ways of dealing with confounds (supplementary Table S8). This is likely due to the fact that the classes for diagnosis classifications (i.e., number of OCD patients and HC) are more balanced across samples.

A number of limitations deserve emphasis. First, we used a sample pooled from existing data across the world, without harmonized protocols for scanning, inclusion criteria or demographic and clinical characteristics. These sources of heterogeneity may limit classification performance, but this also provides an opportunity for model development using independent data sets and the discovery of biomarkers that are reproducible across study sites. Second, standardized FreeSurfer protocols were used for MR data processing to ensure reproducibility across sites. It has been shown that FreeSurfer tends to overestimate subcortical volumes in children^48^, and that MR field strength can affect regional cortical estimations^49^. However, these nonsystematic effects are expected to affect patients and HC equally and are therefore not expected to influence our results. Third, limited information on medication use was available. We were therefore only able to distinguish patients on antidepressants with or without adjuvant antipsychotics vs. those who had not received any medication. Medication history, medication dosage, and duration of use were unknown. Nonetheless, these coarsely defined medication groups enabled better case-control discrimination and good classification of medicated vs. unmedicated cases. Fourth, there is a lack of information on OCD subtypes in our dataset. Particular OCD subtypes may have different neural correlates, and this might limit the ability of MVPA models to find generalizable patterns in brain structure^14,50^. Fifth, it should be noted that the age cut-off used to split the data in pediatric (age below 18) and adult (age 18 and older) samples may not be optimal with respect to the development of the brain, but this was done in accordance with the initial collection of pediatric and adult samples and previous ENIGMA work^51,52^. Finally, it is possible that the brain features used for classification led to sub-optimal performance. OCD is thought to derive from abnormalities distributed at the network-level rather than focused on a single brain area, and FreeSurfer features might not be sufficiently sensitive to detect subtle alterations associated with OCD.

Taken together, this study provides a realistic estimate of the classification performance that can be achieved in a large, ecologically valid, multi-site sample of OCD participants using data on regional brain structure. Our findings show that parcellated structural MRI data do not enable a good overall distinction between patients with OCD and HC. However, classifying subgroups of patients based on medication status enables fair identification at the individual subject level, which implies that medication use is associated with substantial distributed differences in brain anatomy. This underlines the need for longitudinal studies on the short-term and long-term effects of psychiatric medication on brain structure.

## Supplementary information

Supplementary Material

## Data Availability

The computer code for the above-described analyses is publicly available (https://github.com/WillemB2104/ENIGMA-OCD-2020).
