# Supplementary material for: Structural neuroimaging biomarkers for obsessive-compulsive disorder in the ENIGMA-OCD consortium: medication matters
Source: Transl Psychiatry. 2020 Oct 8;10:342. doi: 10.1038/s41398-020-01013-y (PMC7598942; doi:10.1038/s41398-020-01013-y)
Supplement: Supplementary file 1 — Supplementary Material [file 41398_2020_1013_MOESM1_ESM.doc]

**Table of Contents**

[**Supplementary Methods. Details on imputation, multivariate classification and validation and feature importance** 2](#__RefHeading___Toc14781054)

[**Supplementary Tables** 6](#__RefHeading___Toc14781055)

**Supplementary Methods. Details on imputation, multivariate classification and validation, feature importance and clinical variables and sensitivity analyses**

**Imputation benchmark**

Not every subject had complete entries for each feature: out of the total sample containing 4370 participants and 158 FreeSurfer features, 2.56% of data points were missing. The majority of classifier algorithms used in this study cannot deal with missing values, and simply removing those subjects that miss any feature would result in excluding roughly half the subjects in the ENGIMA-OCD sample. For this reason we investigated the feasibility of imputation strategies to replace missing values. This was assessed using simulated data: subjects with complete entries (N=2017 for the complete sample) were used as ground truth to simulate missing values for different fractions of missing data that reflect the distribution of missing values seen in the real dataset. We then apply different imputation techniques and calculated the similarity (normalized root mean square error (NRMSE)) between imputed values and the true values. The following imputation strategies were assessed: median, mean, K-nearest neighbors (KNN), Multiple Imputation by Chained Equations (MICE), Iterative-Singular Value Decomposition (SVD) and SoftImpute implemented using the Python package *fancyimpute* (v. 0.4.0) Results for different imputation strategies using simulated data are summarized in Figure S1. It was found that, for the scope of missing data observed in the ENIGMA-OCD dataset (2.56% missing data points), median, mean, KNN, and MICE imputation all perform equally well. We chose to continue using median imputation as its computational costs are very low, it was found to be reliable across different percentages of missing data, it can be fit on training data and applied on test data separately and is more sensitive to outliers than mean imputation.


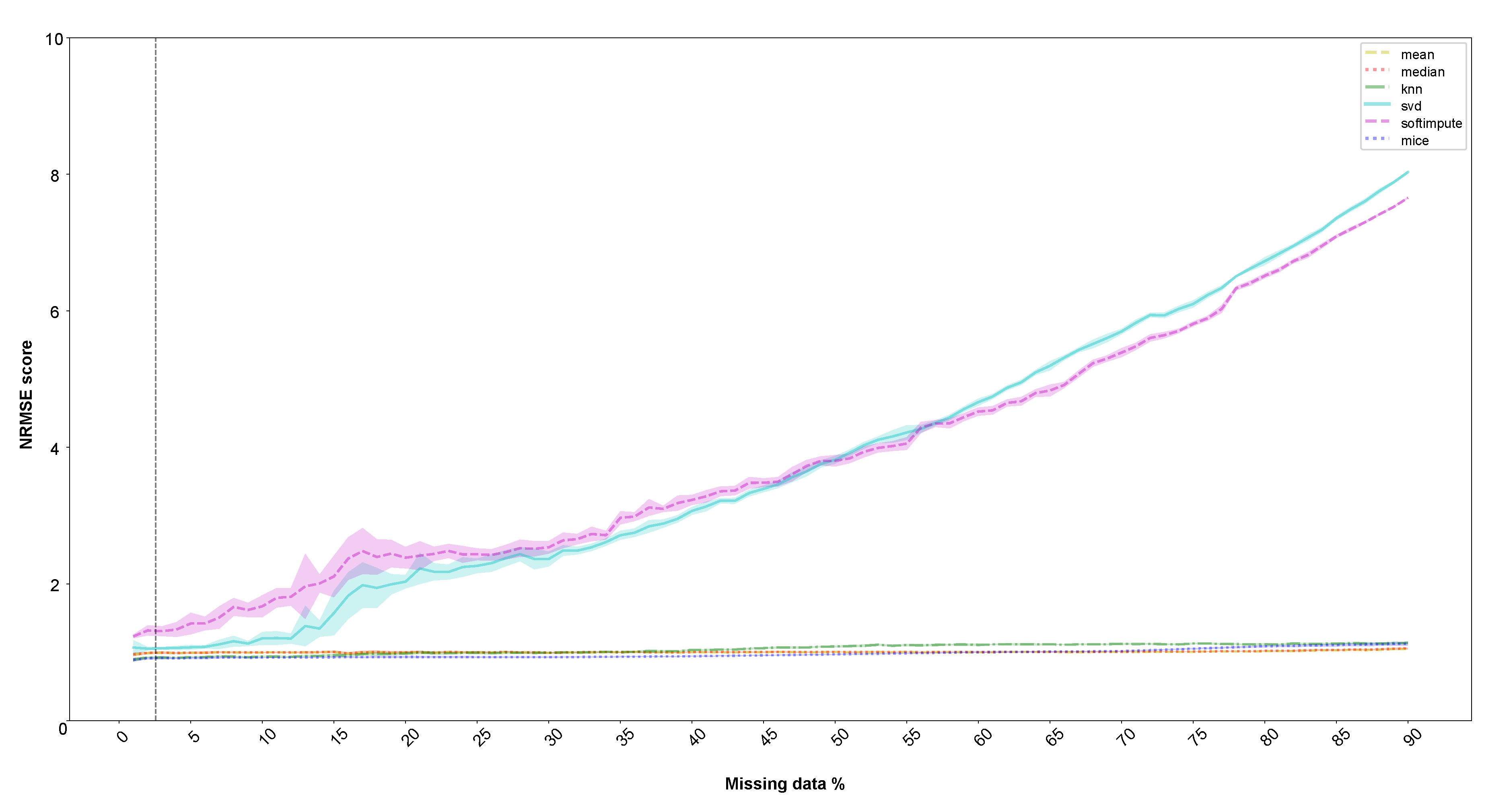


**Figure S1. Similarity between original and imputed data calculated for different fractions of missing values and imputation techniques used.** Similarity is measured in normalized root mean square error (NRMSE); the vertical dashed line indicates the fraction of missing values in the original data (2.56%).

**Multivariate classification and validation**

FreeSurfer variables were combined with covariates age, sex and site by concatenating individual feature vectors. Continuous features were centered around median zero and scaled according to their interquartile range. Median and interquartile range estimations were performed on training data only and applied on test data separately. Categorical covariates were one-hot encoded prior to classification. One-hot encoded variables are referred to as ‘dummy variables’ from hereon.

We assessed multi-site classification using 10-fold site-stratified CV to obtain maximally homogeneous train-test splits, with approximately the same number of subjects in each fold and the same proportion of samples coming from each. Additionally, we addressed leave-one-site-out (LOSO) CV, in which all but one site were used to train the models while the left out site was used to assess model performance. Finally, as LOSO-CV has different fold sizes, we also performed site-stratified CV with varying fold sizes, in which the number of CV folds and test-fold sizes are matched to those of LOSO-CV, to evaluate whether differences in performance were due to differences in heterogeneity or fold size. Here CV-folds are matched to the amount of different sites used for classification, and the test-size within each CV-iteration is set to N samples of each respective site.



**Figure S2. Schematical representation of different cross-validation schemes used for multi-site classification.** Modified from ‘Deriving reproducible biomarkers from multi-site resting-state data: An Autism-based example’, NeuroImage, 147, Abraham, A., Milham, M. P., Di Martino, A., Craddock, R. C., Samaras, D., Thirion, B., & Varoquaux, G., Pages No. 736–745, <https://doi.org/10.1016/j.neuroimage.2016.10.045>, Copyright (2020) with permission from the publisher.

All MVPA analyses were implemented in Python (2.7.14) using the Shogun toolbox (6.1.3) for GPC, imbalanced learn package (0.4.3) for RFC, xgboost (0.81) for XGBoost and the Scikit-Learn toolbox (0.19.1) for all other classifiers. GPC was fitted with a linear kernel and constant mean function, and approximated using logit likelihood and Laplace’s method^1^. Hyperparameters for RFC were chosen following general recommendations for the field: the amount of trees used was set to be as large as computationally feasible with 1000 estimators, and these were fitted using subsampling without replacement to avoid bias in feature importances ^2–4^. Subsample sizes of both classes were set to halve the minority class to ensure balanced class distributions^3,5^. Hyperparameters for SVM (LIBSVM), LR and XGBoost were optimized using nested cross-validation^6^. For both linear and non-linear radial basis function (RBF) kernel SVM, a grid search was performed to tune the C parameter to one of the following values: 0.0001, 0.001, 0.01, 0.1. Additionally, the gamma parameter for RBF SVM was optimized to 0.001, 0.01, 0.1 or 1. For logistic regression, a grid search was performed across both C (set to 0.0001, 0.001, 0.01, 0.1, 1 or 10) and type of regularization penalty used (L1, L2). Hyperparametrization for XGBoost was performed using a grid search across gamma (0.5, 1, 1.5, 2, 5), learning rate (0.01, 0.1, 0.3) and max depth (3, 4, 5). The type of CV used to grid search was set to match that of outer CV: classifications performed using 10-fold site-stratified CV were combined with a grid search using 5-fold site-stratified CV, and for LOGO a Group 5-fold CV (in which multiple sites are left out for testing) was used.

The neural network was constructed using five layers. The first layers consisted of 158 input nodes, followed by hidden layers with 60, 40 and 20 notes respectively, feeding into the last layer consisting of a single node for binary classification. All nodes used the Rectified Linear Units (ReLu) as the activation functions, apart from the last node which used the hyperbolic tangent activation function (to bound model output)^7^. All weights were initialized to random values sampled from a normal distribution with a mean of 0 and a standard deviation of 0.03. Apart from the shallow Neural Network topology, L2 regularization was used in the last hidden layer (with a lambda of 0.0001) to avoid overfitting. The model was optimized using the mean squared error using Adam as the optimization update scheme^8^. All training was performed using 450 epochs, apart from the single site classification for which 250 epochs was used.

Potential bias in estimated model performance is mostly associated with CV performed on small sample sizes (N<100), and given the large size of the ENIGMA-OCD sample and thus the large computational costs, permutation tests to assess statistical significance of model performance were not carried out^9,10^. Instead, we tested significance of model performance directly through obtained AUC scores. Reported AUC scores were computed using the trapezoidal rule (scikit-learn implementation)^11^, and were averaged across CV-folds. The AUC can be interpreted as a sum of ranks and has been shown to be identical to the normalized Mann-Whitney U (and Wilcoxon rank-sum) statistic used for nonparametric testing^12–14^. Given a large enough sample, the significance of the U statistic can be accurately assessed using a normal-distribution approximation. We used this approach to derive a p-value per classifier using the average AUC and average class sizes across CV folds. Similarly, 95% confidence intervals (CI) were computed using the Mann-Whitney U approximation (also known as Hanley-McNeil’s method^13^). It should be noted that the Mann-Whitney U test might lead to overly optimistic results in a CV setting, as its intended use is for testing statistical significance on an independent test set and reported p-values should be interpreted with caution^15^. However, biases in estimations for CV setting are mostly associated with small sample sizes and are not likely to be an issue given our large sample. Additionally, conservative Bonferroni correction for 10 classifiers x 3 CV types was used to control family-wise error.

**Feature importance**

In order to assess which brain and clinical features contributed most to classification we chose to focus on the RFC, as it is able to provide descriptive internal estimates of feature importance. These can however be biased, as this approach tends to assign higher importance values towards continuous predictors that vary in their scale of measurement or categorical features with many categories^3,4,16^. Therefore, we used permutations-based inference and a significance testing framework, in which each feature is permuted separately by shuffling its corresponding values across all subjects so that its original association with its target outcome (e.g. diagnosis) is broken^4,17,18^. This shuffled data is then used to retrain RFC to extract permuted feature importance, and this procedure is repeated 500 times to create an empirical null distribution of importance values per feature. Next we assessed significance for each feature using its corresponding empirical distribution, with false discovery rate (FDR) correction for multiple testing (two stage FDR- correction using Benjamini-Hochberg procedure). Only those predictors with p-values surviving FDR correction in over halve of CV folds are reported, yielding a set of significant and consistent features important for classification. Age, sex and site were introduced as covariates for all analyses, and permutations were performed using scikit and statsmodels for FDR correction. Feature importance’s for dummy encoded variables (i.e. site ID) were shuffled using block-permutations and used to derive one p-value per dummy code for each CV iteration. Tippett’s method was used for joint inference in order obtain a single p-value for site ID (across dummy codes) for each CV iteration; which in turn could be tested for significance using FDR correction as described previously^19^.

**Supplementary Tables**

**Table S1.** Summary of site demographics for each site contributing to the ENIGMA-OCD consortium. Asterisks depict sites that were not included in previous ENIGMA-OCD mega-analyses^20,21^.

| **Site** | **Age Group** | **Tesla** | **Group** | **N** | **Age** | **Male (%)** | **Medicated** | **Onset** | **Severity** |
| --- | --- | --- | --- | --- | --- | --- | --- | --- | --- |
| Beucke | adult | 1.5 | Control | 104 | 31.92 | 51 |  |  |  |
|  |  |  | Patient | 92 | 32.41 | 46 | 55 | 17.18 | 20.07 |
| Cheng | adult | 1.5 | Control | 40 | 31.43 | 13 |  |  |  |
|  |  |  | Patient | 24 | 30.63 | 9 | 7 | 26.83 | 31.00 |
| van den Heuvel | adult | 1.5 | Control | 49 | 31.57 | 19 |  |  |  |
|  |  |  | Patient | 54 | 33.54 | 16 | 54 | 14.41 | 22.71 |
| Hoexter | adult | 1.5 | Control | 37 | 27.62 | 13 |  |  |  |
|  |  |  | Patient | 50 | 31.46 | 22 | 40 | 13.10 | 27.20 |
| KwonNMC | adult | 1.5 | Control | 103 | 24.10 | 57 |  |  |  |
|  |  |  | Patient | 45 | 24.76 | 34 | 34 | 17.44 | 20.22 |
| KwonSNU | adult | 1.5 | Control | 45 | 24.89 | 29 |  |  |  |
|  |  |  | Patient | 41 | 28.10 | 26 | 41 | 18.12 | 23.47 |
| Mataix_Cols | adult | 1.5 | Control | 33 | 36.12 | 12 |  |  |  |
|  |  |  | Patient | 44 | 38.68 | 19 | 14 | 18.39 | 25.86 |
| Menchon | adult | 1.5 | Control | 66 | 33.06 | 30 |  |  |  |
|  |  |  | Patient | 117 | 34.83 | 58 | 4 | 21.44 | 25.50 |
| **Morgado*** | adult | 1.5 | Control | 53 | 27.58 | 20 |  |  |  |
|  |  |  | Patient | 59 | 27.69 | 28 | 13 |  | 25.95 |
| Nakamae | adult | 1.5 | Control | 48 | 30.44 | 22 |  |  |  |
|  |  |  | Patient | 82 | 31.61 | 39 | 43 | 24.73 | 24.83 |
| Reddy | adult | 1.5 | Control | 22 | 26.36 | 15 |  |  |  |
|  |  |  | Patient | 68 | 28.00 | 37 | 68 | 22.13 | 25.68 |
| Benedetti | adult | 3 | Control | 62 | 33.98 | 45 |  |  |  |
|  |  |  | Patient | 66 | 35.02 | 47 | 24 | 16.02 | 30.89 |
| **Brennan*** | adult | 3 | Control | 29 | 32.38 | 13 |  |  |  |
|  |  |  | Patient | 98 | 28.84 | 55 | 14 |  |  |
| Cheng | adult | 3 | Control | 95 | 26.19 | 27 |  |  |  |
|  |  |  | Patient | 56 | 32.89 | 31 | 18 | 27.23 | 28.21 |
| Denys | adult | 3 | Control | 25 | 39.64 | 11 |  |  |  |
|  |  |  | Patient | 31 | 35.26 | 8 | 10 | 18.17 | 27.48 |
| van den Heuvel | adult | 3 | Control | 38 | 39.61 | 18 |  |  |  |
|  |  |  | Patient | 42 | 38.32 | 20 | 42 | 15.49 | 21.48 |
| **Hirano*** | adult | 3 | Control | 44 | 30.95 | 20 |  |  |  |
|  |  |  | Patient | 47 | 33.11 | 17 | 9 | 22.74 | 26.30 |
| Koch | adult | 3 | Control | 74 | 30.27 | 29 |  |  |  |
|  |  |  | Patient | 76 | 30.91 | 28 | 31 | 16.97 | 20.77 |
| Kwon | adult | 3 | Control | 89 | 26.26 | 54 |  |  |  |
|  |  |  | Patient | 90 | 26.70 | 56 | 88 | 19.04 | 26.67 |
| Nakamae | adult | 3 | Control | 42 | 29.57 | 19 |  |  |  |
|  |  |  | Patient | 34 | 32.82 | 12 | 34 | 25.06 | 22.06 |
| Nakao | adult | 3 | Control | 41 | 39.34 | 16 |  |  |  |
|  |  |  | Patient | 81 | 36.60 | 34 | 10 | 24.62 | 22.48 |
| Nurmi | adult | 3 | Control | 25 | 30.76 | 14 |  |  |  |
|  |  |  | Patient | 49 | 33.31 | 25 | 34 | 10.85 | 24.61 |
| Reddy | adult | 3 | Control | 170 | 26.59 | 108 |  |  |  |
|  |  |  | Patient | 203 | 29.50 | 107 | 121 | 22.09 | 25.87 |
| Simpson | adult | 3 | Control | 33 | 28.27 | 17 |  |  |  |
|  |  |  | Patient | 33 | 29.62 | 17 | 33 | 15.03 | 25.55 |
| Spalletta | adult | 3 | Control | 128 | 36.52 | 76 |  |  |  |
|  |  |  | Patient | 84 | 36.67 | 56 | 5 | 18.90 | 23.44 |
| Stein | adult | 3 | Control | 29 | 30.59 | 11 |  |  |  |
|  |  |  | Patient | 23 | 30.48 | 11 | 14 | 14.00 | 23.09 |
| **Stern*** | adult | 3 | Control | 18 | 28.17 | 8 |  |  |  |
|  |  |  | Patient | 15 | 27.87 | 5 | 3 | 12.40 | 18.80 |
| Tolin | adult | 3 | Control | 32 | 48.00 | 7 |  |  |  |
|  |  |  | Patient | 27 | 32.11 | 18 | 4 |  | 22.74 |
| Walitza | adult | 3 | Control | 18 | 32.89 | 5 |  |  |  |
|  |  |  | Patient | 17 | 30.72 | 8 | 6 | 16.71 | 17.12 |
| Wang | adult | 3 | Control | 37 | 26.24 | 20 |  |  |  |
|  |  |  | Patient | 53 | 29.47 | 29 | 53 | 23.19 | 25.34 |
| **Buitelaar*** | pediatric | 1.5 | Control | 61 | 10.93 | 44 |  |  |  |
|  |  |  | Patient | 22 | 10.57 | 14 | 12 |  |  |
| James | pediatric | 1.5 | Control | 12 | 16.63 | 7 |  |  |  |
|  |  |  | Patient | 13 | 16.30 | 7 | 7 | 11.45 | 13.85 |
| Lazaro | pediatric | 1.5 | Control | 32 | 14.63 | 15 |  |  |  |
|  |  |  | Patient | 31 | 14.61 | 18 | 14 | 12.42 | 22.23 |
| Arnold | pediatric | 3 | Control | 13 | 12.30 | 7 |  |  |  |
|  |  |  | Patient | 35 | 12.77 | 21 | 13 | 8.67 | 20.92 |
| Fitzgerald | pediatric | 3 | Control | 59 | 12.96 | 30 |  |  |  |
|  |  |  | Patient | 62 | 14.17 | 30 | 32 | 9.89 | 19.10 |
| Gruner | pediatric | 3 | Control | 23 | 14.19 | 12 |  |  |  |
|  |  |  | Patient | 23 | 14.33 | 13 | 11 |  | 26.87 |
| **Hirano*** | pediatric | 3 | Control | 6 | 15.33 | 4 |  |  |  |
|  |  |  | Patient | 20 | 14.00 | 13 | 12 | 11.90 | 26.89 |
| Hoexter | pediatric | 3 | Control | 29 | 11.93 | 17 |  |  |  |
|  |  |  | Patient | 28 | 12.61 | 17 | 15 | 7.18 | 26.93 |
| Huyser | pediatric | 3 | Control | 25 | 13.32 | 9 |  |  |  |
|  |  |  | Patient | 27 | 13.59 | 10 | 27 | 10.92 | 25.11 |
| Lazaro | pediatric | 3 | Control | 44 | 14.57 | 24 |  |  |  |
|  |  |  | Patient | 58 | 14.57 | 35 | 12 | 12.02 | 18.59 |
| **Marsh*** | pediatric | 3 | Control | 14 | 9.14 | 8 |  |  |  |
|  |  |  | Patient | 25 | 12.12 | 13 | 25 | 9.32 | 24.40 |
| Nurmi | pediatric | 3 | Control | 36 | 13.30 | 18 |  |  |  |
|  |  |  | Patient | 59 | 12.53 | 32 | 51 |  | 24.05 |
| Reddy | pediatric | 3 | Control | 13 | 13.08 | 6 |  |  |  |
|  |  |  | Patient | 20 | 14.75 | 12 | 3 | 13.10 | 23.70 |
| Soreni | pediatric | 3 | Control | 22 | 11.09 | 11 |  |  |  |
|  |  |  | Patient | 36 | 13.14 | 13 | 31 |  | 21.89 |
| **Stewart*** | pediatric | 3 | Control | 30 | 14.02 | 12 |  |  |  |
|  |  |  | Patient | 28 | 15.04 | 11 | 6 | 9.24 | 13.39 |
| Walitza | pediatric | 3 | Control | 20 | 14.64 | 10 |  |  |  |
|  |  |  | Patient | 16 | 15.68 | 13 | 7 | 11.06 | 14.69 |

**Table S2.** Multi-site classification results for combined samples using different classifiers and CV schemes. Reported metrics are averages across CV folds. Asterisks indicate AUC scores significantly different from chance (p<0.05 Bonferroni corrected (10 classifiers x 3 CV types)) and 95% CI are shown in brackets.

| **CV scheme** | | Inter-site CV | Intra-site CV (fixed fold-size) | Intra-site CV (varying fold-size) |
| --- | --- | --- | --- | --- |
| **Classification** | | Diagnosis | Diagnosis | Diagnosis |
| **Age_Group** | | combined | combined | combined |
| **N** | | 3857 | 3857 | 3857 |
| **SVM** | **Balanced Accuracy** | 0.51 | 0.57 | 0.58 |
|  | **AUC** | 0.53 (0.42-0.64) | 0.59 (0.53-0.65)* | 0.61 (0.5-0.72) |
|  | **Sensitivity** | 0.53 | 0.56 | 0.57 |
|  | **Specificity** | 0.49 | 0.58 | 0.59 |
| **PCA + SVM** | **Balanced Accuracy** | 0.52 | 0.56 | 0.57 |
|  | **AUC** | 0.53 (0.42-0.65) | 0.58 (0.52-0.64) | 0.58 (0.47-0.69) |
|  | **Sensitivity** | 0.53 | 0.57 | 0.58 |
|  | **Specificity** | 0.51 | 0.54 | 0.55 |
| **RBF-SVM** | **Balanced Accuracy** | 0.51 | 0.56 | 0.57 |
|  | **AUC** | 0.52 (0.4-0.63) | 0.6 (0.55-0.66)* | 0.61 (0.5-0.72) |
|  | **Sensitivity** | 0.57 | 0.65 | 0.61 |
|  | **Specificity** | 0.44 | 0.46 | 0.54 |
| **PCA + RBF-SVM** | **Balanced Accuracy** | 0.50 | 0.55 | 0.55 |
|  | **AUC** | 0.51 (0.4-0.63) | 0.59 (0.53-0.65)* | 0.6 (0.48-0.71) |
|  | **Sensitivity** | 0.53 | 0.78 | 0.69 |
|  | **Specificity** | 0.48 | 0.32 | 0.42 |
| **LOG** | **Balanced Accuracy** | 0.51 | 0.57 | 0.57 |
|  | **AUC** | 0.53 (0.42-0.64) | 0.6 (0.54-0.66)* | 0.6 (0.49-0.71) |
|  | **Sensitivity** | 0.48 | 0.58 | 0.58 |
|  | **Specificity** | 0.55 | 0.56 | 0.56 |
| **PCA + LOG** | **Balanced Accuracy** | 0.52 | 0.56 | 0.56 |
|  | **AUC** | 0.53 (0.42-0.64) | 0.58 (0.52-0.64) | 0.59 (0.47-0.7) |
|  | **Sensitivity** | 0.50 | 0.56 | 0.56 |
|  | **Specificity** | 0.53 | 0.56 | 0.56 |
| **GPC** | **Balanced Accuracy** | 0.52 | 0.57 | 0.57 |
|  | **AUC** | 0.53 (0.41-0.64) | 0.59 (0.53-0.64)* | 0.59 (0.48-0.7) |
|  | **Sensitivity** | 0.51 | 0.57 | 0.58 |
|  | **Specificity** | 0.53 | 0.56 | 0.55 |
| **BRFC** | **Balanced Accuracy** | 0.51 | 0.59 | 0.58 |
|  | **AUC** | 0.54 (0.42-0.65) | 0.62 (0.56-0.67)* | 0.62 (0.51-0.73) |
|  | **Sensitivity** | 0.49 | 0.57 | 0.57 |
|  | **Specificity** | 0.53 | 0.60 | 0.59 |
| **XGB** | **Balanced Accuracy** | 0.51 | 0.57 | 0.56 |
|  | **AUC** | 0.52 (0.41-0.63) | 0.6 (0.54-0.65)* | 0.6 (0.49-0.71) |
|  | **Sensitivity** | 0.55 | 0.62 | 0.62 |
|  | **Specificity** | 0.47 | 0.52 | 0.51 |
| **NN** | **Balanced Accuracy** | 0.50 | 0.54 | 0.54 |
|  | **AUC** | 0.51 (0.4-0.62) | 0.57 (0.51-0.63) | 0.56 (0.45-0.67) |
|  | **Sensitivity** | 0.56 | 0.61 | 0.60 |
|  | **Specificity** | 0.44 | 0.48 | 0.48 |

**Table S3.** Multi-site classification results for pediatric samples using different classifiers and CV schemes. Reported metrics are averages across CV folds. Asterisks indicate AUC scores significantly different from chance (p<0.05 Bonferroni corrected (10 classifiers x 3 CV types)) and 95% CI are shown in brackets.

| **CV scheme** | | Inter-site CV | Intra-site CV (fixed fold-size) | Intra-site CV (varying fold-size) |
| --- | --- | --- | --- | --- |
| **Classification** | | Diagnosis | Diagnosis | Diagnosis |
| **Age_Group** | | pediatric | pediatric | pediatric |
| **N** | | 870 | 870 | 870 |
| **SVM** | **Balanced Accuracy** | 0.52 | 0.58 | 0.56 |
|  | **AUC** | 0.52 (0.37-0.67) | 0.6 (0.48-0.72) | 0.6 (0.45-0.75) |
|  | **Sensitivity** | 0.54 | 0.61 | 0.6 |
|  | **Specificity** | 0.51 | 0.55 | 0.53 |
| **PCA + SVM** | **Balanced Accuracy** | 0.52 | 0.58 | 0.58 |
|  | **AUC** | 0.53 (0.38-0.68) | 0.61 (0.49-0.73) | 0.61 (0.46-0.76) |
|  | **Sensitivity** | 0.60 | 0.60 | 0.6 |
|  | **Specificity** | 0.43 | 0.56 | 0.56 |
| **RBF-SVM** | **Balanced Accuracy** | 0.54 | 0.58 | 0.59 |
|  | **AUC** | 0.53 (0.38-0.68) | 0.61 (0.49-0.73) | 0.6 (0.46-0.75) |
|  | **Sensitivity** | 0.64 | 0.73 | 0.68 |
|  | **Specificity** | 0.44 | 0.44 | 0.5 |
| **PCA + RBF-SVM** | **Balanced Accuracy** | 0.53 | 0.57 | 0.58 |
|  | **AUC** | 0.53 (0.38-0.68) | 0.58 (0.46-0.7) | 0.61 (0.46-0.76) |
|  | **Sensitivity** | 0.64 | 0.70 | 0.65 |
|  | **Specificity** | 0.42 | 0.45 | 0.51 |
| **LOG** | **Balanced Accuracy** | 0.49 | 0.59 | 0.57 |
|  | **AUC** | 0.51 (0.36-0.66) | 0.62 (0.5-0.73) | 0.61 (0.46-0.75) |
|  | **Sensitivity** | 0.49 | 0.58 | 0.59 |
|  | **Specificity** | 0.50 | 0.61 | 0.56 |
| **PCA + LOG** | **Balanced Accuracy** | 0.50 | 0.57 | 0.58 |
|  | **AUC** | 0.53 (0.38-0.68) | 0.61 (0.49-0.72) | 0.59 (0.45-0.74) |
|  | **Sensitivity** | 0.59 | 0.58 | 0.58 |
|  | **Specificity** | 0.41 | 0.56 | 0.58 |
| **GPC** | **Balanced Accuracy** | 0.54 | 0.60 | 0.58 |
|  | **AUC** | 0.55 (0.4-0.69) | 0.62 (0.51-0.74) | 0.63 (0.48-0.77) |
|  | **Sensitivity** | 0.60 | 0.63 | 0.62 |
|  | **Specificity** | 0.48 | 0.56 | 0.54 |
| **BRFC** | **Balanced Accuracy** | 0.54 | 0.59 | 0.6 |
|  | **AUC** | 0.53 (0.38-0.68) | 0.62 (0.5-0.74) | 0.62 (0.48-0.77) |
|  | **Sensitivity** | 0.64 | 0.63 | 0.65 |
|  | **Specificity** | 0.44 | 0.55 | 0.55 |
| **XGB** | **Balanced Accuracy** | 0.54 | 0.58 | 0.58 |
|  | **AUC** | 0.56 (0.41-0.71) | 0.6 (0.48-0.72) | 0.61 (0.46-0.76) |
|  | **Sensitivity** | 0.65 | 0.66 | 0.64 |
|  | **Specificity** | 0.44 | 0.50 | 0.53 |
| **NN** | **Balanced Accuracy** | 0.50 | 0.55 | 0.56 |
|  | **AUC** | 0.49 (0.34-0.64) | 0.55 (0.43-0.67) | 0.57 (0.42-0.72) |
|  | **Sensitivity** | 0.62 | 0.65 | 0.66 |
|  | **Specificity** | 0.38 | 0.44 | 0.46 |

**Table S4. Multi-site classification results for adult samples using different classifiers and CV schemes. Reported metrics are averages across CV folds. Asterisks indicate AUC scores significantly different from chance (p<0.05 Bonferroni corrected (10 classifiers x 3 CV types)) and 95% CI are shown in brackets.**

| **CV scheme** | | Inter-site CV | Intra-site CV (fixed fold-size) | Intra-site CV (varying fold-size) |
| --- | --- | --- | --- | --- |
| **Classification** | | Diagnosis | Diagnosis | Diagnosis |
| **Age_Group** | | adult | adult | adult |
| **N** | | 3219 | 3219 | 3219 |
| **SVM** | **Balanced Accuracy** | 0.52 | 0.56 | 0.56 |
|  | **AUC** | 0.53 (0.42-0.64) | 0.57 (0.51-0.64) | 0.59 (0.48-0.7) |
|  | **Sensitivity** | 0.50 | 0.53 | 0.53 |
|  | **Specificity** | 0.55 | 0.58 | 0.59 |
| **PCA + SVM** | **Balanced Accuracy** | 0.51 | 0.56 | 0.56 |
|  | **AUC** | 0.52 (0.41-0.63) | 0.58 (0.52-0.64) | 0.59 (0.48-0.7) |
|  | **Sensitivity** | 0.49 | 0.52 | 0.53 |
|  | **Specificity** | 0.54 | 0.60 | 0.59 |
| **RBF-SVM** | **Balanced Accuracy** | 0.51 | 0.55 | 0.54 |
|  | **AUC** | 0.51 (0.4-0.62) | 0.6 (0.54-0.66)* | 0.59 (0.48-0.7) |
|  | **Sensitivity** | 0.70 | 0.68 | 0.69 |
|  | **Specificity** | 0.31 | 0.41 | 0.38 |
| **PCA + RBF-SVM** | **Balanced Accuracy** | 0.51 | 0.55 | 0.54 |
|  | **AUC** | 0.51 (0.4-0.62) | 0.58 (0.52-0.65) | 0.59 (0.49-0.7) |
|  | **Sensitivity** | 0.63 | 0.73 | 0.79 |
|  | **Specificity** | 0.38 | 0.36 | 0.29 |
| **LOG** | **Balanced Accuracy** | 0.50 | 0.55 | 0.57 |
|  | **AUC** | 0.52 (0.41-0.63) | 0.58 (0.52-0.64) | 0.6 (0.49-0.71) |
|  | **Sensitivity** | 0.50 | 0.53 | 0.55 |
|  | **Specificity** | 0.51 | 0.57 | 0.59 |
| **PCA + LOG** | **Balanced Accuracy** | 0.51 | 0.56 | 0.56 |
|  | **AUC** | 0.51 (0.41-0.62) | 0.58 (0.51-0.64) | 0.59 (0.48-0.7) |
|  | **Sensitivity** | 0.52 | 0.55 | 0.56 |
|  | **Specificity** | 0.50 | 0.57 | 0.56 |
| **GPC** | **Balanced Accuracy** | 0.51 | 0.56 | 0.57 |
|  | **AUC** | 0.52 (0.41-0.63) | 0.59 (0.53-0.65) | 0.6 (0.49-0.7) |
|  | **Sensitivity** | 0.52 | 0.56 | 0.57 |
|  | **Specificity** | 0.51 | 0.56 | 0.57 |
| **BRFC** | **Balanced Accuracy** | 0.51 | 0.57 | 0.58 |
|  | **AUC** | 0.53 (0.42-0.64) | 0.61 (0.55-0.67)* | 0.62 (0.51-0.73) |
|  | **Sensitivity** | 0.59 | 0.61 | 0.58 |
|  | **Specificity** | 0.43 | 0.53 | 0.59 |
| **XGB** | **Balanced Accuracy** | 0.51 | 0.57 | 0.57 |
|  | **AUC** | 0.53 (0.42-0.64) | 0.61 (0.55-0.67)* | 0.61 (0.51-0.72) |
|  | **Sensitivity** | 0.59 | 0.61 | 0.62 |
|  | **Specificity** | 0.43 | 0.53 | 0.53 |
| **NN** | **Balanced Accuracy** | 0.51 | 0.55 | 0.54 |
|  | **AUC** | 0.51 (0.4-0.62) | 0.56 (0.5-0.62) | 0.57 |
|  | **Sensitivity** | 0.53 | 0.58 | 0.58 |
|  | **Specificity** | 0.48 | 0.51 | 0.51 |

**Table S5. Model performances for OCD diagnosis classification performed in single-sites separately using repeated stratified 5-fold cross-validation with 10 repeats. Reported metrics are averages across CV folds and repeats.**

| **Site** | **Tesla** | **Age_Group** | **N_sample** | **SVM** | | | | **PCA + SVM** | | | | **RBF-SVM** | | | | **PCA + RBF-SVM** | | | | **LOG** | | | | **PCA + LOG** | | | | **GPC** | | | | **BRFC** | | | | **XGB** | | | | **NN** | | | |
| --- | --- | --- | --- | --- | --- | --- | --- | --- | --- | --- | --- | --- | --- | --- | --- | --- | --- | --- | --- | --- | --- | --- | --- | --- | --- | --- | --- | --- | --- | --- | --- | --- | --- | --- | --- | --- | --- | --- | --- | --- | --- | --- | --- |
|  |  |  |  | **Balanced Accuracy** | **AUC** | **Sensitivity** | **Specificity** | **Balanced Accuracy** | **AUC** | **Sensitivity** | **Specificity** | **Balanced Accuracy** | **AUC** | **Sensitivity** | **Specificity** | **Balanced Accuracy** | **AUC** | **Sensitivity** | **Specificity** | **Balanced Accuracy** | **AUC** | **Sensitivity** | **Specificity** | **Balanced Accuracy** | **AUC** | **Sensitivity** | **Specificity** | **Balanced Accuracy** | **AUC** | **Sensitivity** | **Specificity** | **Balanced Accuracy** | **AUC** | **Sensitivity** | **Specificity** | **Balanced Accuracy** | **AUC** | **Sensitivity** | **Specificity** | **Balanced Accuracy** | **AUC** | **Sensitivity** | **Specificity** |
| Beucke | 1.5 | adult | 183 | 0.49 | 0.48 | 0.86 | 0.12 | 0.49 | 0.48 | 0.89 | 0.08 | 0.51 | 0.48 | 0.79 | 0.23 | 0.51 | 0.49 | 0.68 | 0.33 | 0.49 | 0.49 | 0.35 | 0.63 | 0.50 | 0.50 | 0.38 | 0.62 | 0.45 | 0.45 | 0.27 | 0.63 | 0.56 | 0.60 | 0.54 | 0.58 | 0.60 | 0.64 | 0.44 | 0.75 | 0.44 | 0.42 | 0.31 | 0.56 |
| Cheng | 1.5 | adult | 56 | 0.51 | 0.40 | 0.40 | 0.63 | 0.52 | 0.41 | 0.43 | 0.62 | 0.50 | 0.44 | 0.27 | 0.74 | 0.50 | 0.44 | 0.28 | 0.71 | 0.55 | 0.57 | 0.52 | 0.58 | 0.52 | 0.58 | 0.43 | 0.61 | 0.52 | 0.57 | 0.39 | 0.65 | 0.53 | 0.56 | 0.49 | 0.58 | 0.49 | 0.48 | 0.24 | 0.74 | 0.43 | 0.48 | 0.52 | 0.34 |
| van den Heuvel | 1.5 | adult | 96 | 0.52 | 0.45 | 0.77 | 0.26 | 0.52 | 0.45 | 0.79 | 0.24 | 0.50 | 0.47 | 0.86 | 0.14 | 0.50 | 0.48 | 0.85 | 0.15 | 0.54 | 0.53 | 0.51 | 0.57 | 0.53 | 0.52 | 0.52 | 0.54 | 0.57 | 0.57 | 0.54 | 0.59 | 0.53 | 0.53 | 0.50 | 0.56 | 0.50 | 0.50 | 0.53 | 0.48 | 0.48 | 0.51 | 0.48 | 0.47 |
| Hoexter | 1.5 | adult | 86 | 0.67 | 0.72 | 0.74 | 0.59 | 0.69 | 0.75 | 0.75 | 0.63 | 0.60 | 0.49 | 0.78 | 0.42 | 0.60 | 0.58 | 0.78 | 0.43 | 0.67 | 0.72 | 0.72 | 0.61 | 0.69 | 0.74 | 0.73 | 0.65 | 0.68 | 0.75 | 0.76 | 0.60 | 0.62 | 0.68 | 0.66 | 0.58 | 0.56 | 0.61 | 0.67 | 0.44 | 0.64 | 0.66 | 0.80 | 0.47 |
| KwonNMC | 1.5 | adult | 148 | 0.51 | 0.39 | 0.56 | 0.46 | 0.51 | 0.40 | 0.60 | 0.43 | 0.51 | 0.46 | 0.55 | 0.47 | 0.51 | 0.43 | 0.49 | 0.53 | 0.54 | 0.57 | 0.52 | 0.56 | 0.53 | 0.58 | 0.51 | 0.55 | 0.49 | 0.56 | 0.20 | 0.79 | 0.55 | 0.58 | 0.52 | 0.58 | 0.48 | 0.51 | 0.12 | 0.85 | 0.48 | 0.56 | 0.04 | 0.91 |
| KwonSNU | 1.5 | adult | 86 | 0.50 | 0.47 | 0.44 | 0.56 | 0.50 | 0.46 | 0.45 | 0.55 | 0.51 | 0.50 | 0.41 | 0.62 | 0.52 | 0.47 | 0.46 | 0.58 | 0.48 | 0.47 | 0.34 | 0.63 | 0.49 | 0.50 | 0.36 | 0.62 | 0.51 | 0.53 | 0.47 | 0.56 | 0.49 | 0.51 | 0.46 | 0.53 | 0.52 | 0.53 | 0.47 | 0.57 | 0.41 | 0.45 | 0.36 | 0.46 |
| Mataix | 1.5 | adult | 74 | 0.60 | 0.53 | 0.67 | 0.52 | 0.56 | 0.58 | 0.63 | 0.49 | 0.60 | 0.52 | 0.80 | 0.39 | 0.56 | 0.52 | 0.87 | 0.25 | 0.57 | 0.56 | 0.60 | 0.54 | 0.55 | 0.56 | 0.57 | 0.53 | 0.55 | 0.57 | 0.71 | 0.39 | 0.57 | 0.63 | 0.63 | 0.51 | 0.58 | 0.62 | 0.69 | 0.47 | 0.59 | 0.68 | 0.84 | 0.33 |
| Menchon | 1.5 | adult | 181 | 0.61 | 0.67 | 0.69 | 0.52 | 0.62 | 0.68 | 0.68 | 0.55 | 0.58 | 0.60 | 0.73 | 0.43 | 0.59 | 0.60 | 0.70 | 0.47 | 0.62 | 0.68 | 0.67 | 0.58 | 0.64 | 0.71 | 0.67 | 0.60 | 0.62 | 0.69 | 0.81 | 0.43 | 0.62 | 0.66 | 0.62 | 0.61 | 0.57 | 0.67 | 0.84 | 0.31 | 0.63 | 0.73 | 0.80 | 0.46 |
| Morgado | 1.5 | adult | 112 | 0.55 | 0.52 | 0.55 | 0.55 | 0.53 | 0.48 | 0.48 | 0.58 | 0.56 | 0.55 | 0.51 | 0.60 | 0.56 | 0.54 | 0.44 | 0.68 | 0.57 | 0.59 | 0.54 | 0.61 | 0.57 | 0.60 | 0.57 | 0.58 | 0.60 | 0.61 | 0.56 | 0.64 | 0.56 | 0.59 | 0.47 | 0.65 | 0.57 | 0.60 | 0.57 | 0.57 | 0.55 | 0.62 | 0.61 | 0.48 |
| Nakamae | 1.5 | adult | 129 | 0.57 | 0.53 | 0.66 | 0.47 | 0.60 | 0.58 | 0.64 | 0.56 | 0.54 | 0.47 | 0.83 | 0.25 | 0.54 | 0.47 | 0.81 | 0.27 | 0.62 | 0.63 | 0.63 | 0.61 | 0.64 | 0.65 | 0.59 | 0.69 | 0.59 | 0.64 | 0.76 | 0.42 | 0.61 | 0.63 | 0.61 | 0.62 | 0.51 | 0.53 | 0.74 | 0.29 | 0.58 | 0.64 | 0.72 | 0.44 |
| Reddy | 1.5 | adult | 51 | 0.50 | 0.50 | 0.41 | 0.59 | 0.49 | 0.49 | 0.52 | 0.46 | 0.50 | 0.39 | 0.27 | 0.73 | 0.51 | 0.43 | 0.33 | 0.69 | 0.49 | 0.44 | 0.38 | 0.59 | 0.45 | 0.42 | 0.27 | 0.62 | 0.51 | 0.49 | 0.64 | 0.38 | 0.48 | 0.48 | 0.47 | 0.49 | 0.48 | 0.48 | 0.66 | 0.30 | 0.43 | 0.44 | 0.71 | 0.15 |
| Benedetti | 3 | adult | 53 | 0.56 | 0.52 | 0.41 | 0.71 | 0.59 | 0.60 | 0.47 | 0.71 | 0.54 | 0.37 | 0.32 | 0.76 | 0.57 | 0.45 | 0.40 | 0.74 | 0.60 | 0.63 | 0.58 | 0.63 | 0.60 | 0.64 | 0.57 | 0.64 | 0.61 | 0.65 | 0.57 | 0.66 | 0.63 | 0.68 | 0.60 | 0.66 | 0.61 | 0.64 | 0.59 | 0.62 | 0.59 | 0.72 | 0.78 | 0.40 |
| Brennan | 3 | adult | 124 | 0.83 | 0.89 | 0.83 | 0.83 | 0.81 | 0.88 | 0.83 | 0.79 | 0.72 | 0.80 | 0.87 | 0.57 | 0.77 | 0.80 | 0.85 | 0.70 | 0.77 | 0.83 | 0.76 | 0.79 | 0.79 | 0.85 | 0.79 | 0.79 | 0.69 | 0.83 | 0.85 | 0.54 | 0.69 | 0.77 | 0.69 | 0.70 | 0.62 | 0.78 | 0.92 | 0.31 | 0.52 | 0.69 | 1.00 | 0.03 |
| Cheng | 3 | adult | 137 | 0.69 | 0.74 | 0.62 | 0.75 | 0.65 | 0.70 | 0.54 | 0.75 | 0.65 | 0.71 | 0.61 | 0.69 | 0.64 | 0.68 | 0.58 | 0.69 | 0.71 | 0.76 | 0.67 | 0.75 | 0.68 | 0.73 | 0.64 | 0.71 | 0.68 | 0.76 | 0.53 | 0.83 | 0.64 | 0.72 | 0.62 | 0.66 | 0.65 | 0.73 | 0.45 | 0.86 | 0.67 | 0.73 | 0.65 | 0.70 |
| Denys | 3 | adult | 39 | 0.45 | 0.43 | 0.57 | 0.33 | 0.53 | 0.53 | 0.54 | 0.52 | 0.48 | 0.57 | 0.90 | 0.06 | 0.46 | 0.58 | 0.79 | 0.13 | 0.46 | 0.48 | 0.36 | 0.56 | 0.48 | 0.50 | 0.38 | 0.58 | 0.49 | 0.48 | 0.53 | 0.45 | 0.44 | 0.46 | 0.43 | 0.44 | 0.68 | 0.69 | 0.56 | 0.80 | 0.55 | 0.56 | 0.70 | 0.40 |
| van den Heuvel | 3 | adult | 77 | 0.45 | 0.54 | 0.60 | 0.31 | 0.43 | 0.61 | 0.54 | 0.33 | 0.49 | 0.58 | 0.80 | 0.18 | 0.50 | 0.54 | 0.88 | 0.12 | 0.45 | 0.44 | 0.23 | 0.67 | 0.45 | 0.43 | 0.12 | 0.78 | 0.40 | 0.37 | 0.48 | 0.33 | 0.38 | 0.33 | 0.38 | 0.38 | 0.43 | 0.41 | 0.51 | 0.35 | 0.48 | 0.51 | 0.48 | 0.47 |
| Hirano | 3 | adult | 91 | 0.52 | 0.50 | 0.55 | 0.49 | 0.50 | 0.46 | 0.47 | 0.53 | 0.48 | 0.49 | 0.50 | 0.47 | 0.48 | 0.50 | 0.55 | 0.42 | 0.56 | 0.60 | 0.57 | 0.55 | 0.49 | 0.52 | 0.48 | 0.51 | 0.54 | 0.57 | 0.57 | 0.51 | 0.54 | 0.57 | 0.56 | 0.53 | 0.50 | 0.50 | 0.49 | 0.51 | 0.53 | 0.58 | 0.67 | 0.38 |
| Koch | 3 | adult | 146 | 0.50 | 0.48 | 0.37 | 0.63 | 0.50 | 0.48 | 0.32 | 0.68 | 0.49 | 0.51 | 0.17 | 0.80 | 0.48 | 0.51 | 0.31 | 0.65 | 0.48 | 0.50 | 0.47 | 0.49 | 0.48 | 0.50 | 0.46 | 0.51 | 0.50 | 0.52 | 0.48 | 0.53 | 0.53 | 0.53 | 0.51 | 0.54 | 0.58 | 0.61 | 0.59 | 0.57 | 0.58 | 0.56 | 0.57 | 0.59 |
| Kwon | 3 | adult | 179 | 0.52 | 0.51 | 0.59 | 0.46 | 0.54 | 0.51 | 0.58 | 0.50 | 0.51 | 0.46 | 0.76 | 0.27 | 0.52 | 0.45 | 0.80 | 0.24 | 0.60 | 0.64 | 0.60 | 0.60 | 0.52 | 0.53 | 0.48 | 0.55 | 0.55 | 0.55 | 0.56 | 0.53 | 0.67 | 0.75 | 0.59 | 0.74 | 0.68 | 0.74 | 0.65 | 0.71 | 0.55 | 0.58 | 0.60 | 0.51 |
| Nakamae | 3 | adult | 73 | 0.45 | 0.55 | 0.59 | 0.31 | 0.48 | 0.46 | 0.50 | 0.46 | 0.49 | 0.57 | 0.79 | 0.20 | 0.49 | 0.55 | 0.67 | 0.31 | 0.44 | 0.40 | 0.20 | 0.67 | 0.47 | 0.48 | 0.36 | 0.59 | 0.41 | 0.40 | 0.36 | 0.46 | 0.45 | 0.44 | 0.42 | 0.48 | 0.51 | 0.53 | 0.43 | 0.60 | 0.47 | 0.48 | 0.49 | 0.46 |
| Nakao | 3 | adult | 120 | 0.60 | 0.65 | 0.75 | 0.46 | 0.60 | 0.62 | 0.72 | 0.48 | 0.52 | 0.48 | 0.86 | 0.18 | 0.51 | 0.47 | 0.88 | 0.13 | 0.58 | 0.62 | 0.68 | 0.47 | 0.58 | 0.61 | 0.65 | 0.51 | 0.55 | 0.60 | 0.83 | 0.26 | 0.57 | 0.59 | 0.63 | 0.51 | 0.52 | 0.54 | 0.85 | 0.20 | 0.51 | 0.53 | 0.94 | 0.07 |
| Nurmi | 3 | adult | 72 | 0.51 | 0.45 | 0.47 | 0.55 | 0.51 | 0.45 | 0.45 | 0.56 | 0.49 | 0.49 | 0.50 | 0.49 | 0.50 | 0.48 | 0.58 | 0.41 | 0.50 | 0.50 | 0.50 | 0.51 | 0.50 | 0.52 | 0.49 | 0.51 | 0.52 | 0.53 | 0.73 | 0.32 | 0.56 | 0.58 | 0.56 | 0.56 | 0.49 | 0.49 | 0.83 | 0.15 | 0.51 | 0.52 | 0.73 | 0.30 |
| Reddy | 3 | adult | 371 | 0.62 | 0.65 | 0.63 | 0.61 | 0.62 | 0.66 | 0.61 | 0.63 | 0.62 | 0.66 | 0.56 | 0.68 | 0.63 | 0.65 | 0.58 | 0.67 | 0.62 | 0.66 | 0.60 | 0.64 | 0.63 | 0.68 | 0.64 | 0.62 | 0.63 | 0.68 | 0.65 | 0.60 | 0.64 | 0.69 | 0.61 | 0.66 | 0.64 | 0.69 | 0.70 | 0.57 | 0.57 | 0.60 | 0.63 | 0.51 |
| Simpson | 3 | adult | 66 | 0.49 | 0.49 | 0.47 | 0.50 | 0.47 | 0.49 | 0.42 | 0.53 | 0.49 | 0.52 | 0.50 | 0.49 | 0.49 | 0.53 | 0.50 | 0.49 | 0.49 | 0.48 | 0.43 | 0.55 | 0.44 | 0.43 | 0.29 | 0.59 | 0.45 | 0.47 | 0.49 | 0.41 | 0.41 | 0.38 | 0.39 | 0.43 | 0.44 | 0.41 | 0.41 | 0.46 | 0.46 | 0.51 | 0.60 | 0.33 |
| Spalletta | 3 | adult | 207 | 0.61 | 0.61 | 0.59 | 0.63 | 0.60 | 0.62 | 0.62 | 0.59 | 0.64 | 0.67 | 0.61 | 0.67 | 0.60 | 0.62 | 0.54 | 0.66 | 0.60 | 0.64 | 0.60 | 0.61 | 0.61 | 0.64 | 0.59 | 0.63 | 0.62 | 0.68 | 0.46 | 0.79 | 0.61 | 0.65 | 0.58 | 0.63 | 0.58 | 0.62 | 0.42 | 0.75 | 0.62 | 0.67 | 0.57 | 0.67 |
| Stein | 3 | adult | 49 | 0.53 | 0.42 | 0.51 | 0.55 | 0.51 | 0.44 | 0.50 | 0.53 | 0.50 | 0.56 | 0.44 | 0.56 | 0.47 | 0.55 | 0.37 | 0.58 | 0.48 | 0.48 | 0.34 | 0.62 | 0.46 | 0.46 | 0.34 | 0.59 | 0.53 | 0.56 | 0.41 | 0.64 | 0.46 | 0.44 | 0.40 | 0.53 | 0.45 | 0.39 | 0.36 | 0.53 | 0.56 | 0.61 | 0.57 | 0.55 |
| Stern | 3 | adult | 31 | 0.47 | 0.52 | 0.21 | 0.74 | 0.47 | 0.52 | 0.25 | 0.68 | 0.50 | 0.66 | 0.20 | 0.80 | 0.50 | 0.56 | 0.26 | 0.74 | 0.47 | 0.50 | 0.25 | 0.69 | 0.45 | 0.44 | 0.11 | 0.78 | 0.42 | 0.36 | 0.22 | 0.62 | 0.42 | 0.42 | 0.35 | 0.50 | 0.52 | 0.54 | 0.36 | 0.69 | 0.78 | 0.76 | 0.90 | 0.65 |
| Tolin | 3 | adult | 59 | 0.59 | 0.50 | 0.66 | 0.52 | 0.62 | 0.61 | 0.60 | 0.64 | 0.62 | 0.54 | 0.68 | 0.56 | 0.64 | 0.57 | 0.68 | 0.61 | 0.71 | 0.75 | 0.66 | 0.77 | 0.71 | 0.76 | 0.65 | 0.77 | 0.75 | 0.78 | 0.68 | 0.82 | 0.74 | 0.75 | 0.67 | 0.80 | 0.66 | 0.70 | 0.60 | 0.73 | 0.67 | 0.72 | 0.70 | 0.63 |
| Walitza | 3 | adult | 35 | 0.48 | 0.43 | 0.48 | 0.48 | 0.49 | 0.41 | 0.46 | 0.53 | 0.51 | 0.46 | 0.41 | 0.60 | 0.51 | 0.45 | 0.42 | 0.61 | 0.49 | 0.45 | 0.42 | 0.55 | 0.52 | 0.51 | 0.48 | 0.55 | 0.56 | 0.56 | 0.52 | 0.59 | 0.57 | 0.56 | 0.56 | 0.59 | 0.45 | 0.45 | 0.33 | 0.57 | 0.59 | 0.64 | 0.62 | 0.56 |
| Wang | 3 | adult | 88 | 0.47 | 0.48 | 0.47 | 0.47 | 0.48 | 0.49 | 0.49 | 0.47 | 0.49 | 0.49 | 0.40 | 0.58 | 0.49 | 0.52 | 0.43 | 0.55 | 0.46 | 0.46 | 0.37 | 0.54 | 0.50 | 0.50 | 0.43 | 0.57 | 0.46 | 0.47 | 0.61 | 0.30 | 0.48 | 0.49 | 0.48 | 0.49 | 0.45 | 0.42 | 0.65 | 0.25 | 0.44 | 0.49 | 0.67 | 0.22 |
| Buitelaar | 1.5 | pediatric | 77 | 0.54 | 0.49 | 0.28 | 0.81 | 0.52 | 0.50 | 0.23 | 0.81 | 0.52 | 0.49 | 0.27 | 0.78 | 0.52 | 0.52 | 0.28 | 0.75 | 0.47 | 0.44 | 0.14 | 0.79 | 0.48 | 0.48 | 0.15 | 0.80 | 0.50 | 0.40 | 0.09 | 0.92 | 0.45 | 0.40 | 0.32 | 0.57 | 0.50 | 0.52 | 0.07 | 0.92 | 0.48 | 0.51 | 0.20 | 0.76 |
| Lazaro | 1.5 | pediatric | 61 | 0.53 | 0.46 | 0.61 | 0.46 | 0.58 | 0.45 | 0.58 | 0.58 | 0.55 | 0.47 | 0.76 | 0.34 | 0.53 | 0.48 | 0.65 | 0.40 | 0.54 | 0.55 | 0.52 | 0.57 | 0.60 | 0.61 | 0.52 | 0.68 | 0.61 | 0.64 | 0.59 | 0.62 | 0.60 | 0.62 | 0.52 | 0.67 | 0.48 | 0.52 | 0.51 | 0.46 | 0.54 | 0.52 | 0.68 | 0.40 |
| Arnold | 3 | pediatric | 48 | 0.41 | 0.61 | 0.66 | 0.16 | 0.44 | 0.58 | 0.67 | 0.22 | 0.49 | 0.59 | 0.97 | 0.00 | 0.50 | 0.55 | 0.95 | 0.05 | 0.45 | 0.41 | 0.18 | 0.72 | 0.46 | 0.42 | 0.14 | 0.77 | 0.45 | 0.31 | 0.84 | 0.06 | 0.40 | 0.35 | 0.45 | 0.34 | 0.47 | 0.39 | 0.93 | 0.02 | 0.53 | 0.35 | 0.86 | 0.20 |
| Fitzgerald | 3 | pediatric | 113 | 0.50 | 0.52 | 0.53 | 0.47 | 0.50 | 0.51 | 0.54 | 0.46 | 0.50 | 0.49 | 0.47 | 0.53 | 0.49 | 0.50 | 0.58 | 0.40 | 0.46 | 0.46 | 0.41 | 0.51 | 0.48 | 0.47 | 0.44 | 0.52 | 0.52 | 0.53 | 0.53 | 0.51 | 0.52 | 0.52 | 0.52 | 0.52 | 0.54 | 0.55 | 0.57 | 0.52 | 0.45 | 0.44 | 0.47 | 0.42 |
| Gruner | 3 | pediatric | 44 | 0.53 | 0.47 | 0.41 | 0.65 | 0.47 | 0.55 | 0.34 | 0.60 | 0.53 | 0.46 | 0.18 | 0.88 | 0.53 | 0.52 | 0.26 | 0.80 | 0.48 | 0.47 | 0.37 | 0.59 | 0.46 | 0.43 | 0.24 | 0.67 | 0.46 | 0.42 | 0.36 | 0.56 | 0.47 | 0.49 | 0.42 | 0.51 | 0.54 | 0.57 | 0.50 | 0.59 | 0.57 | 0.59 | 0.77 | 0.37 |
| Hoexter | 3 | pediatric | 55 | 0.55 | 0.50 | 0.44 | 0.67 | 0.55 | 0.49 | 0.47 | 0.63 | 0.53 | 0.44 | 0.39 | 0.67 | 0.53 | 0.43 | 0.35 | 0.71 | 0.57 | 0.57 | 0.54 | 0.59 | 0.56 | 0.56 | 0.53 | 0.58 | 0.55 | 0.62 | 0.56 | 0.53 | 0.57 | 0.61 | 0.49 | 0.65 | 0.54 | 0.55 | 0.55 | 0.53 | 0.60 | 0.59 | 0.75 | 0.45 |
| Huyser | 3 | pediatric | 47 | 0.53 | 0.57 | 0.58 | 0.49 | 0.61 | 0.70 | 0.65 | 0.57 | 0.50 | 0.49 | 0.43 | 0.56 | 0.51 | 0.52 | 0.48 | 0.54 | 0.54 | 0.63 | 0.55 | 0.54 | 0.61 | 0.70 | 0.59 | 0.63 | 0.55 | 0.63 | 0.53 | 0.58 | 0.53 | 0.57 | 0.52 | 0.54 | 0.51 | 0.49 | 0.56 | 0.45 | 0.54 | 0.71 | 0.85 | 0.23 |
| Lazaro | 3 | pediatric | 102 | 0.77 | 0.85 | 0.77 | 0.77 | 0.79 | 0.84 | 0.80 | 0.78 | 0.75 | 0.82 | 0.74 | 0.77 | 0.75 | 0.82 | 0.70 | 0.79 | 0.76 | 0.84 | 0.76 | 0.76 | 0.78 | 0.85 | 0.76 | 0.80 | 0.76 | 0.82 | 0.76 | 0.76 | 0.73 | 0.79 | 0.73 | 0.74 | 0.71 | 0.79 | 0.77 | 0.66 | 0.70 | 0.80 | 0.77 | 0.64 |
| Marsh | 3 | pediatric | 39 | 0.49 | 0.32 | 0.23 | 0.75 | 0.49 | 0.33 | 0.28 | 0.70 | 0.49 | 0.37 | 0.21 | 0.78 | 0.49 | 0.37 | 0.22 | 0.77 | 0.61 | 0.64 | 0.60 | 0.61 | 0.58 | 0.64 | 0.56 | 0.60 | 0.58 | 0.66 | 0.72 | 0.44 | 0.60 | 0.64 | 0.63 | 0.57 | 0.54 | 0.58 | 0.80 | 0.29 | 0.49 | 0.43 | 0.73 | 0.25 |
| Nurmi | 3 | pediatric | 92 | 0.52 | 0.45 | 0.63 | 0.41 | 0.51 | 0.46 | 0.60 | 0.42 | 0.50 | 0.53 | 0.45 | 0.55 | 0.50 | 0.49 | 0.60 | 0.39 | 0.54 | 0.53 | 0.58 | 0.49 | 0.49 | 0.48 | 0.42 | 0.56 | 0.52 | 0.53 | 0.68 | 0.35 | 0.55 | 0.56 | 0.55 | 0.55 | 0.55 | 0.58 | 0.77 | 0.34 | 0.51 | 0.52 | 0.73 | 0.30 |
| Reddy | 3 | pediatric | 26 | 0.56 | 0.38 | 0.83 | 0.29 | 0.55 | 0.35 | 0.81 | 0.29 | 0.52 | 0.33 | 0.78 | 0.26 | 0.50 | 0.30 | 0.75 | 0.26 | 0.66 | 0.70 | 0.68 | 0.64 | 0.67 | 0.72 | 0.65 | 0.68 | 0.69 | 0.72 | 0.76 | 0.62 | 0.65 | 0.74 | 0.68 | 0.63 | 0.68 | 0.71 | 0.79 | 0.58 | 0.57 | 0.60 | 0.63 | 0.51 |
| Soreni | 3 | pediatric | 47 | 0.58 | 0.58 | 0.66 | 0.50 | 0.61 | 0.62 | 0.69 | 0.53 | 0.54 | 0.42 | 0.44 | 0.63 | 0.52 | 0.47 | 0.39 | 0.64 | 0.60 | 0.64 | 0.61 | 0.59 | 0.63 | 0.65 | 0.65 | 0.60 | 0.63 | 0.67 | 0.70 | 0.56 | 0.59 | 0.64 | 0.61 | 0.57 | 0.54 | 0.58 | 0.64 | 0.44 | 0.66 | 0.71 | 0.85 | 0.47 |
| Stewart | 3 | pediatric | 57 | 0.50 | 0.44 | 0.62 | 0.38 | 0.50 | 0.45 | 0.55 | 0.46 | 0.49 | 0.51 | 0.58 | 0.40 | 0.49 | 0.50 | 0.62 | 0.37 | 0.47 | 0.49 | 0.39 | 0.54 | 0.49 | 0.52 | 0.42 | 0.57 | 0.48 | 0.53 | 0.41 | 0.55 | 0.49 | 0.50 | 0.51 | 0.48 | 0.50 | 0.51 | 0.48 | 0.53 | 0.43 | 0.43 | 0.57 | 0.30 |
| Walitza | 3 | pediatric | 36 | 0.54 | 0.46 | 0.38 | 0.70 | 0.49 | 0.47 | 0.31 | 0.68 | 0.49 | 0.51 | 0.19 | 0.79 | 0.50 | 0.53 | 0.33 | 0.67 | 0.51 | 0.49 | 0.39 | 0.64 | 0.54 | 0.50 | 0.41 | 0.67 | 0.58 | 0.56 | 0.46 | 0.70 | 0.49 | 0.49 | 0.45 | 0.54 | 0.44 | 0.39 | 0.31 | 0.56 | 0.59 | 0.64 | 0.62 | 0.56 |

**Table S6a.** Classifications performed in subsamples of OCD patients stratified for age of onset. Reported metrics are averages across CV folds. Asterisks indicate AUC scores significantly different from chance (p<0.05 Bonferroni corrected (10 classifiers x 3 comparisons)) and 95% CI are shown in brackets.

| **CV scheme** | | Intra-site CV (fixed fold-size) | Intra-site CV (fixed fold-size) | Intra-site CV (fixed fold-size)) |
| --- | --- | --- | --- | --- |
| **Classification** | | Age of Onset | Diagnosis | Diagnosis |
| **Filter** | | None | Early Onset | Late Onset |
| **Age_Group** | | combined | combined | combined |
| **N** | | 1534 | 2677 | 2436 |
| **SVM** | **Balanced Accuracy** | 0.69 | 0.63 | 0.64 |
|  | **AUC** | 0.77 (0.7-0.84)* | 0.67 (0.61-0.74)* | 0.69 (0.62-0.76)* |
|  | **Sensitivity** | 0.73 | 0.62 | 0.65 |
|  | **Specificity** | 0.65 | 0.64 | 0.62 |
| **PCA + SVM** | **Balanced Accuracy** | 0.69 | 0.58 | 0.63 |
|  | **AUC** | 0.76 (0.68-0.83)* | 0.61 (0.54-0.68)* | 0.69 (0.62-0.75)* |
|  | **Sensitivity** | 0.75 | 0.56 | 0.66 |
|  | **Specificity** | 0.64 | 0.6 | 0.61 |
| **RBF-SVM** | **Balanced Accuracy** | 0.7 | 0.6 | 0.66 |
|  | **AUC** | 0.77 (0.7-0.85)* | 0.64 (0.57-0.71)* | 0.71 (0.65-0.78)* |
|  | **Sensitivity** | 0.75 | 0.53 | 0.63 |
|  | **Specificity** | 0.65 | 0.67 | 0.68 |
| **PCA + RBF-SVM** | **Balanced Accuracy** | 0.68 | 0.59 | 0.65 |
|  | **AUC** | 0.76 (0.68-0.83)* | 0.63 (0.56-0.69)* | 0.71 (0.64-0.77)* |
|  | **Sensitivity** | 0.74 | 0.52 | 0.65 |
|  | **Specificity** | 0.62 | 0.65 | 0.65 |
| **LOG** | **Balanced Accuracy** | 0.72 | 0.62 | 0.64 |
|  | **AUC** | 0.79 (0.72-0.86)* | 0.67 (0.61-0.74)* | 0.7 (0.63-0.76)* |
|  | **Sensitivity** | 0.76 | 0.61 | 0.64 |
|  | **Specificity** | 0.68 | 0.64 | 0.64 |
| **PCA + LOG** | **Balanced Accuracy** | 0.69 | 0.58 | 0.64 |
|  | **AUC** | 0.76 (0.69-0.84)* | 0.61 (0.54-0.68) | 0.69 (0.63-0.76)* |
|  | **Sensitivity** | 0.73 | 0.56 | 0.66 |
|  | **Specificity** | 0.65 | 0.59 | 0.62 |
| **GPC** | **Balanced Accuracy** | 0.71 | 0.56 | 0.62 |
|  | **AUC** | 0.78 (0.71-0.85)* | 0.62 (0.55-0.69)* | 0.69 (0.62-0.75)* |
|  | **Sensitivity** | 0.73 | 0.3 | 0.44 |
|  | **Specificity** | 0.69 | 0.83 | 0.8 |
| **BRFC** | **Balanced Accuracy** | 0.72 | 0.56 | 0.6 |
|  | **AUC** | 0.81 (0.74-0.88)* | 0.68 (0.62-0.75)* | 0.73 (0.67-0.79)* |
|  | **Sensitivity** | 0.78 | 0.21 | 0.32 |
|  | **Specificity** | 0.65 | 0.91 | 0.88 |
| **XGB** | **Balanced Accuracy** | 0.69 | 0.6 | 0.65 |
|  | **AUC** | 0.78 (0.71-0.85)* | 0.64 (0.58-0.71)* | 0.71 (0.64-0.77)* |
|  | **Sensitivity** | 0.73 | 0.61 | 0.69 |
|  | **Specificity** | 0.65 | 0.59 | 0.61 |
| **NN** | **Balanced Accuracy** | 0.64 | 0.52 | 0.60 |
|  | **AUC** | 0.69 (0.61-0.77)* | 0.55 (0.48-0.62) | 0.68 (0.61-0.75)* |
|  | **Sensitivity** | 0.60 | 0.32 | 0.41 |
|  | **Specificity** | 0.68 | 0.72 | 0.78 |

**Table S6b.** Classifications performed in subsamples of OCD patients stratified for disease duration. Reported metrics are averages across CV folds. Asterisks indicate AUC scores significantly different from chance (p<0.05 Bonferroni corrected (10 classifiers x 3 comparisons)) and 95% CI are shown in brackets.

| **CV scheme** | | Intra-site CV (fixed fold-size) | Intra-site CV (fixed fold-size) | Intra-site CV (fixed fold-size)) |
| --- | --- | --- | --- | --- |
| **Classification** | | Duration | Diagnosis | Diagnosis |
| **Filter** | | None | Low Duration | High Duration |
| **Age_Group** | | combined | combined | combined |
| **N** | | 1535 | 2549 | 2550 |
| **SVM** | **Balanced Accuracy** | 0.67 | 0.61 | 0.63 |
|  | **AUC** | 0.75 (0.67-0.83)* | 0.66 (0.6-0.73)* | 0.68 (0.62-0.75)* |
|  | **Sensitivity** | 0.66 | 0.6 | 0.64 |
|  | **Specificity** | 0.69 | 0.62 | 0.63 |
| **PCA + SVM** | **Balanced Accuracy** | 0.67 | 0.58 | 0.63 |
|  | **AUC** | 0.73 (0.65-0.81)* | 0.61 (0.54-0.68) | 0.68 (0.61-0.74)* |
|  | **Sensitivity** | 0.64 | 0.6 | 0.62 |
|  | **Specificity** | 0.69 | 0.56 | 0.64 |
| **RBF-SVM** | **Balanced Accuracy** | 0.68 | 0.6 | 0.63 |
|  | **AUC** | 0.74 (0.66-0.82)* | 0.64 (0.57-0.71)* | 0.69 (0.63-0.76)* |
|  | **Sensitivity** | 0.66 | 0.55 | 0.59 |
|  | **Specificity** | 0.7 | 0.64 | 0.66 |
| **PCA + RBF-SVM** | **Balanced Accuracy** | 0.66 | 0.57 | 0.63 |
|  | **AUC** | 0.73 (0.65-0.81)* | 0.61 (0.54-0.69)* | 0.68 (0.62-0.75)* |
|  | **Sensitivity** | 0.65 | 0.47 | 0.61 |
|  | **Specificity** | 0.66 | 0.68 | 0.65 |
| **LOG** | **Balanced Accuracy** | 0.69 | 0.61 | 0.64 |
|  | **AUC** | 0.77 (0.69-0.84)* | 0.67 (0.61-0.74)* | 0.7 (0.64-0.76)* |
|  | **Sensitivity** | 0.68 | 0.61 | 0.64 |
|  | **Specificity** | 0.71 | 0.62 | 0.64 |
| **PCA + LOG** | **Balanced Accuracy** | 0.66 | 0.58 | 0.63 |
|  | **AUC** | 0.73 (0.65-0.81)* | 0.62 (0.54-0.69)* | 0.68 (0.62-0.75)* |
|  | **Sensitivity** | 0.65 | 0.59 | 0.63 |
|  | **Specificity** | 0.67 | 0.58 | 0.64 |
| **GPC** | **Balanced Accuracy** | 0.68 | 0.56 | 0.6 |
|  | **AUC** | 0.75 (0.67-0.82)* | 0.62 (0.55-0.69)* | 0.67 (0.61-0.74)* |
|  | **Sensitivity** | 0.68 | 0.29 | 0.4 |
|  | **Specificity** | 0.67 | 0.83 | 0.8 |
| **BRFC** | **Balanced Accuracy** | 0.69 | 0.56 | 0.57 |
|  | **AUC** | 0.78 (0.7-0.85)* | 0.68 (0.61-0.75)* | 0.71 (0.65-0.78)* |
|  | **Sensitivity** | 0.73 | 0.2 | 0.25 |
|  | **Specificity** | 0.65 | 0.92 | 0.9 |
| **XGB** | **Balanced Accuracy** | 0.68 | 0.61 | 0.63 |
|  | **AUC** | 0.75 (0.67-0.82)* | 0.65 (0.58-0.72)* | 0.68 (0.61-0.75)* |
|  | **Sensitivity** | 0.71 | 0.69 | 0.67 |
|  | **Specificity** | 0.64 | 0.54 | 0.6 |
| NN | **Balanced Accuracy** | 0.61 | 0.54 | 0.57 |
|  | **AUC** | 0.66 (0.57-0.75)* | 0.60 (0.53-0.67) | 0.62 (0.55-0.69)* |
|  | **Sensitivity** | 0.61 | 0.29 | 0.38 |
|  | **Specificity** | 0.61 | 0.80 | 0.75 |

**Table S6c.** Classifications performed in subsamples of OCD patients stratified for medication status. Reported metrics are averages across CV folds. Asterisks indicate AUC scores significantly different from chance (p<0.05 Bonferroni corrected (10 classifiers x 3 comparisons)) and 95% CI are shown in brackets.

| **CV scheme** | | Intra-site CV (fixed fold-size) | Intra-site CV (fixed fold-size) | Intra-site CV (fixed fold-size)) |
| --- | --- | --- | --- | --- |
| **Classification** | | Medication | Diagnosis | Diagnosis |
| **Filter** | | None | Unmedicated | Medicated |
| **Age_Group** | | combined | combined | combined |
| **N** | | 1745 | 2797 | 2692 |
| **SVM** | **Balanced Accuracy** | 0.76 | 0.62 | 0.64 |
|  | **AUC** | 0.83 (0.77-0.89)* | 0.65 (0.59-0.72)* | 0.71 (0.65-0.78)* |
|  | **Sensitivity** | 0.77 | 0.67 | 0.64 |
|  | **Specificity** | 0.76 | 0.57 | 0.65 |
| **PCA + SVM** | **Balanced Accuracy** | 0.67 | 0.54 | 0.62 |
|  | **AUC** | 0.73 (0.66-0.8)* | 0.56 (0.49-0.63) | 0.66 (0.6-0.73)* |
|  | **Sensitivity** | 0.65 | 0.59 | 0.61 |
|  | **Specificity** | 0.69 | 0.5 | 0.63 |
| **RBF-SVM** | **Balanced Accuracy** | 0.72 | 0.54 | 0.66 |
|  | **AUC** | 0.78 (0.71-0.85)* | 0.59 (0.52-0.66) | 0.72 (0.66-0.78)* |
|  | **Sensitivity** | 0.72 | 0.32 | 0.63 |
|  | **Specificity** | 0.73 | 0.76 | 0.69 |
| **PCA + RBF-SVM** | **Balanced Accuracy** | 0.69 | 0.5 | 0.64 |
|  | **AUC** | 0.75 (0.68-0.82)* | 0.61 (0.54-0.68)* | 0.69 (0.63-0.76)* |
|  | **Sensitivity** | 0.68 | 0.06 | 0.61 |
|  | **Specificity** | 0.69 | 0.95 | 0.68 |
| **LOG** | **Balanced Accuracy** | 0.77 | 0.61 | 0.66 |
|  | **AUC** | 0.85 (0.8-0.91)* | 0.66 (0.59-0.72)* | 0.73 (0.67-0.79)* |
|  | **Sensitivity** | 0.77 | 0.64 | 0.66 |
|  | **Specificity** | 0.76 | 0.59 | 0.65 |
| **PCA + LOG** | **Balanced Accuracy** | 0.68 | 0.54 | 0.62 |
|  | **AUC** | 0.73 (0.66-0.81)* | 0.57 (0.5-0.64) | 0.66 (0.6-0.73)* |
|  | **Sensitivity** | 0.66 | 0.56 | 0.61 |
|  | **Specificity** | 0.7 | 0.53 | 0.63 |
| **GPC** | **Balanced Accuracy** | 0.7 | 0.55 | 0.61 |
|  | **AUC** | 0.77 (0.7-0.84)* | 0.59 (0.52-0.66) | 0.69 (0.63-0.75)* |
|  | **Sensitivity** | 0.65 | 0.31 | 0.4 |
|  | **Specificity** | 0.75 | 0.79 | 0.82 |
| **BRFC** | **Balanced Accuracy** | 0.76 | 0.56 | 0.63 |
|  | **AUC** | 0.86 (0.8-0.91)* | 0.6 (0.54-0.67) | 0.69 (0.63-0.75)* |
|  | **Sensitivity** | 0.73 | 0.52 | 0.53 |
|  | **Specificity** | 0.8 | 0.6 | 0.73 |
| **XGB** | **Balanced Accuracy** | 0.71 | 0.54 | 0.62 |
|  | **AUC** | 0.78 (0.71-0.85)* | 0.61 (0.55-0.68)* | 0.73 (0.67-0.79)* |
|  | **Sensitivity** | 0.68 | 0.21 | 0.35 |
|  | **Specificity** | 0.74 | 0.86 | 0.89 |
| **NN** | **Balanced Accuracy** | 0.64 | 0.54 | 0.58 |
|  | **AUC** | 0.68 (0.6-0.76)* | 0.57 (0.5-0.64) | 0.62 (0.55-0.69)* |
|  | **Sensitivity** | 0.64 | 0.38 | 0.44 |
|  | **Specificity** | 0.63 | 0.69 | 0.71 |

**Table S6d.** Classifications performed in subsamples of OCD patients stratified for severity. Reported metrics are averages across CV folds. Asterisks indicate AUC scores significantly different from chance (p<0.05 Bonferroni corrected (10 classifiers x 3 comparisons)) and 95% CI are shown in brackets.

| **CV scheme** | | Intra-site CV (fixed fold-size) | Intra-site CV (fixed fold-size) | Intra-site CV (fixed fold-size)) |
| --- | --- | --- | --- | --- |
| **Classification** | | Severity | Diagnosis | Diagnosis |
| **Filter** | | None | Low Severity | High Severity |
| **Age_Group** | | combined | combined | combined |
| **N** | | 1630 | 2609 | 2641 |
| **SVM** | **Balanced Accuracy** | 0.57 | 0.59 | 0.59 |
|  | **AUC** | 0.61 (0.52-0.69) | 0.62 (0.55-0.69)* | 0.63 (0.56-0.7)* |
|  | **Sensitivity** | 0.59 | 0.6 | 0.61 |
|  | **Specificity** | 0.56 | 0.59 | 0.57 |
| **PCA + SVM** | **Balanced Accuracy** | 0.56 | 0.53 | 0.58 |
|  | **AUC** | 0.58 (0.49-0.67) | 0.56 (0.49-0.63) | 0.61 (0.54-0.68) |
|  | **Sensitivity** | 0.61 | 0.52 | 0.62 |
|  | **Specificity** | 0.51 | 0.55 | 0.55 |
| **RBF-SVM** | **Balanced Accuracy** | 0.59 | 0.56 | 0.58 |
|  | **AUC** | 0.61 (0.53-0.7) | 0.6 (0.53-0.67) | 0.62 (0.55-0.69)* |
|  | **Sensitivity** | 0.61 | 0.41 | 0.49 |
|  | **Specificity** | 0.57 | 0.7 | 0.67 |
| **PCA + RBF-SVM** | **Balanced Accuracy** | 0.59 | 0.52 | 0.57 |
|  | **AUC** | 0.6 (0.51-0.69) | 0.57 (0.5-0.64) | 0.62 (0.55-0.69)* |
|  | **Sensitivity** | 0.61 | 0.11 | 0.45 |
|  | **Specificity** | 0.57 | 0.93 | 0.69 |
| **LOG** | **Balanced Accuracy** | 0.58 | 0.59 | 0.6 |
|  | **AUC** | 0.61 (0.53-0.7) | 0.62 (0.55-0.69)* | 0.64 (0.57-0.71)* |
|  | **Sensitivity** | 0.59 | 0.58 | 0.59 |
|  | **Specificity** | 0.57 | 0.61 | 0.6 |
| **PCA + LOG** | **Balanced Accuracy** | 0.56 | 0.53 | 0.58 |
|  | **AUC** | 0.58 (0.49-0.67) | 0.56 (0.49-0.63) | 0.61 (0.54-0.68) |
|  | **Sensitivity** | 0.58 | 0.52 | 0.59 |
|  | **Specificity** | 0.55 | 0.54 | 0.56 |
| **GPC** | **Balanced Accuracy** | 0.57 | 0.54 | 0.55 |
|  | **AUC** | 0.59 (0.5-0.67) | 0.6 (0.53-0.67) | 0.6 (0.53-0.67) |
|  | **Sensitivity** | 0.58 | 0.24 | 0.29 |
|  | **Specificity** | 0.55 | 0.85 | 0.82 |
| **BRFC** | **Balanced Accuracy** | 0.55 | 0.52 | 0.55 |
|  | **AUC** | 0.58 (0.49-0.66) | 0.6 (0.53-0.67) | 0.61 (0.54-0.68)* |
|  | **Sensitivity** | 0.54 | 0.15 | 0.19 |
|  | **Specificity** | 0.57 | 0.9 | 0.92 |
| **XGB** | **Balanced Accuracy** | 0.57 | 0.58 | 0.57 |
|  | **AUC** | 0.59 (0.5-0.68) | 0.6 (0.53-0.67) | 0.61 (0.54-0.68) |
|  | **Sensitivity** | 0.57 | 0.64 | 0.61 |
|  | **Specificity** | 0.58 | 0.52 | 0.53 |
| **NN** | **Balanced Accuracy** | 0.53 | 0.52 | 0.54 |
|  | **AUC** | 0.54 (0.45-0.63) | 0.54 (0.47-0.61) | 0.56 (0.49-0.63.) |
|  | **Sensitivity** | 0.57 | 0.26 | 0.33 |
|  | **Specificity** | 0.48 | 0.79 | 0.74 |

**Table S7a.** Sensitivity analyses for unmedicated OCD versus controls classification. Reported metrics are averages across CV folds. Asterisks indicate AUC scores significantly different from chance (p<0.05 Bonferroni corrected (10 classifiers x 2 comparisons)) and 95% CI are shown in brackets.

| **CV scheme** | | Intra-site CV | Intra-site CV |
| --- | --- | --- | --- |
|  |  | (fixed fold-size) | (fixed fold-size) |
| **Classification** | | Unmedicated + Low Duration OCD vs Controls | Unmedicated + High Duration OCD vs Controls |
| **Age_Group** | | combined | combined |
| **N** | | 2175 | 2042 |
| **SVM** | **Balanced Accuracy** | 0.63 | 0.69 |
|  | **AUC** | 0.69 (0.61-0.76)* | 0.73 (0.65-0.81)* |
|  | **Sensitivity** | 0.63 | 0.69 |
|  | **Specificity** | 0.63 | 0.68 |
| **PCA + SVM** | **Balanced Accuracy** | 0.61 | 0.6 |
|  | **AUC** | 0.65 (0.56-0.73)* | 0.66 (0.57-0.74)* |
|  | **Sensitivity** | 0.64 | 0.61 |
|  | **Specificity** | 0.58 | 0.6 |
| **RBF-SVM** | **Balanced Accuracy** | 0.6 | 0.64 |
|  | **AUC** | 0.67 (0.58-0.75)* | 0.7 (0.62-0.78)* |
|  | **Sensitivity** | 0.52 | 0.54 |
|  | **Specificity** | 0.69 | 0.74 |
| **PCA + RBF-SVM** | **Balanced Accuracy** | 0.59 | 0.63 |
|  | **AUC** | 0.65 (0.56-0.73)* | 0.68 (0.6-0.77)* |
|  | **Sensitivity** | 0.49 | 0.6 |
|  | **Specificity** | 0.69 | 0.67 |
| **LOG** | **Balanced Accuracy** | 0.63 | 0.67 |
|  | **AUC** | 0.7 (0.63-0.78)* | 0.74 (0.66-0.82)* |
|  | **Sensitivity** | 0.6 | 0.65 |
|  | **Specificity** | 0.66 | 0.69 |
| **PCA + LOG** | **Balanced Accuracy** | 0.61 | 0.61 |
|  | **AUC** | 0.66 (0.58-0.74)* | 0.66 (0.57-0.74)* |
|  | **Sensitivity** | 0.63 | 0.61 |
|  | **Specificity** | 0.59 | 0.61 |
| **GPC** | **Balanced Accuracy** | 0.55 | 0.55 |
|  | **AUC** | 0.63 (0.54-0.71) | 0.62 (0.52-0.71) |
|  | **Sensitivity** | 0.22 | 0.18 |
|  | **Specificity** | 0.88 | 0.91 |
| **BRFC** | **Balanced Accuracy** | 0.53 | 0.52 |
|  | **AUC** | 0.71 (0.63-0.78)* | 0.71 (0.63-0.79)* |
|  | **Sensitivity** | 0.08 | 0.07 |
|  | **Specificity** | 0.98 | 0.98 |
| **XGB** | **Balanced Accuracy** | 0.58 | 0.63 |
|  | **AUC** | 0.64 (0.56-0.72)* | 0.68 (0.59-0.76)* |
|  | **Sensitivity** | 0.66 | 0.71 |
|  | **Specificity** | 0.51 | 0.56 |
| **NN** | **Balanced Accuracy** | 0.56 | 0.55 |
|  | **AUC** | 0.60 (0.51-0.69) | 0.60 (0.51-0.69) |
|  | **Sensitivity** | 0.30 | 0.23 |
|  | **Specificity** | 0.82 | 0.86 |

**Table S7b.** Sensitivity analyses for medicated OCD vs controls classification. Reported metrics are averages across CV folds. Asterisks indicate AUC scores significantly different from chance (p<0.05 Bonferroni corrected (10 classifiers x 2 comparisons)) and 95% CI are shown in brackets.

| **CV scheme** | | Intra-site CV | Intra-site CV |
| --- | --- | --- | --- |
|  |  | (fixed fold-size) | (fixed fold-size) |
| **Classification** | | Medicated + Low Duration OCD vs Controls | Medicated + High Duration OCD vs Controls |
| **Age_Group** | | combined | combined |
| **N** | | 1994 | 2093 |
| **SVM** | **Balanced Accuracy** | 0.68 | 0.69 |
|  | **AUC** | 0.77 (0.69-0.84)* | 0.76 (0.69-0.83)* |
|  | **Sensitivity** | 0.64 | 0.66 |
|  | **Specificity** | 0.72 | 0.72 |
| **PCA + SVM** | **Balanced Accuracy** | 0.61 | 0.68 |
|  | **AUC** | 0.65 (0.55-0.74) | 0.74 (0.66-0.81)* |
|  | **Sensitivity** | 0.58 | 0.66 |
|  | **Specificity** | 0.63 | 0.7 |
| **RBF-SVM** | **Balanced Accuracy** | 0.63 | 0.68 |
|  | **AUC** | 0.72 (0.63-0.8)* | 0.77 (0.7-0.84)* |
|  | **Sensitivity** | 0.44 | 0.56 |
|  | **Specificity** | 0.82 | 0.8 |
| **PCA + RBF-SVM** | **Balanced Accuracy** | 0.62 | 0.68 |
|  | **AUC** | 0.69 (0.6-0.78)* | 0.75 (0.67-0.82)* |
|  | **Sensitivity** | 0.49 | 0.6 |
|  | **Specificity** | 0.76 | 0.76 |
| **LOG** | **Balanced Accuracy** | 0.69 | 0.71 |
|  | **AUC** | 0.78 (0.71-0.85)* | 0.78 (0.72-0.85)* |
|  | **Sensitivity** | 0.63 | 0.67 |
|  | **Specificity** | 0.75 | 0.75 |
| **PCA + LOG** | **Balanced Accuracy** | 0.61 | 0.69 |
|  | **AUC** | 0.65 (0.56-0.75) | 0.75 (0.67-0.82)* |
|  | **Sensitivity** | 0.58 | 0.68 |
|  | **Specificity** | 0.64 | 0.7 |
| **GPC** | **Balanced Accuracy** | 0.56 | 0.63 |
|  | **AUC** | 0.65 (0.55-0.75) | 0.74 (0.66-0.81)* |
|  | **Sensitivity** | 0.19 | 0.37 |
|  | **Specificity** | 0.92 | 0.88 |
| **BRFC** | **Balanced Accuracy** | 0.55 | 0.58 |
|  | **AUC** | 0.71 (0.62-0.8)* | 0.78 (0.72-0.85)* |
|  | **Sensitivity** | 0.11 | 0.18 |
|  | **Specificity** | 0.99 | 0.97 |
| **XGB** | **Balanced Accuracy** | 0.64 | 0.67 |
|  | **AUC** | 0.68 (0.59-0.77)* | 0.73 (0.66-0.81)* |
|  | **Sensitivity** | 0.68 | 0.69 |
|  | **Specificity** | 0.6 | 0.64 |
| **NN** | **Balanced Accuracy** | 0.52 | 0.59 |
|  | **AUC** | 0.57 (0.47-0.67) | 0.67 (0.58-0.76)* |
|  | **Sensitivity** | 0.14 | 0.29 |
|  | **Specificity** | 0.89 | 0.90 |

**Table S7c.** Sensitivity analyses for medication status classification. Reported metrics are averages across CV folds. Asterisks indicate AUC scores significantly different from chance (p<0.05 Bonferroni corrected (10 classifiers x 2 comparisons)) and 95% CI are shown in brackets.

| **CV scheme** | | Intra-site CV | Intra-site CV |
| --- | --- | --- | --- |
|  |  | (fixed fold-size) | (fixed fold-size) |
| **Classification** | | Unmedicated vs Medicated OCD in Low Duration patients | Unmedicated vs Medicated OCD in High Duration patients |
| **Age_Group** | | combined | combined |
| **N** | | 711 | 738 |
| **SVM** | **Balanced Accuracy** | 0.67 | 0.74 |
|  | **AUC** | 0.73 (0.62-0.85)* | 0.82 (0.72-0.91)* |
|  | **Sensitivity** | 0.64 | 0.74 |
|  | **Specificity** | 0.7 | 0.74 |
| **PCA + SVM** | **Balanced Accuracy** | 0.6 | 0.68 |
|  | **AUC** | 0.64 (0.51-0.77) | 0.74 (0.62-0.85)* |
|  | **Sensitivity** | 0.58 | 0.65 |
|  | **Specificity** | 0.62 | 0.7 |
| **RBF-SVM** | **Balanced Accuracy** | 0.66 | 0.73 |
|  | **AUC** | 0.74 (0.63-0.85)* | 0.79 (0.69-0.9)* |
|  | **Sensitivity** | 0.6 | 0.72 |
|  | **Specificity** | 0.72 | 0.73 |
| **PCA + RBF-SVM** | **Balanced Accuracy** | 0.67 | 0.7 |
|  | **AUC** | 0.73 (0.62-0.85)* | 0.78 (0.68-0.89)* |
|  | **Sensitivity** | 0.63 | 0.65 |
|  | **Specificity** | 0.71 | 0.75 |
| **LOG** | **Balanced Accuracy** | 0.68 | 0.76 |
|  | **AUC** | 0.76 (0.65-0.87)* | 0.85 (0.76-0.94)* |
|  | **Sensitivity** | 0.64 | 0.74 |
|  | **Specificity** | 0.71 | 0.78 |
| **PCA + LOG** | **Balanced Accuracy** | 0.62 | 0.68 |
|  | **AUC** | 0.65 (0.52-0.78) | 0.74 (0.63-0.85)* |
|  | **Sensitivity** | 0.63 | 0.68 |
|  | **Specificity** | 0.61 | 0.68 |
| **GPC** | **Balanced Accuracy** | 0.63 | 0.7 |
|  | **AUC** | 0.68 (0.56-0.8) | 0.77 (0.66-0.88)* |
|  | **Sensitivity** | 0.54 | 0.69 |
|  | **Specificity** | 0.72 | 0.72 |
| **BRFC** | **Balanced Accuracy** | 0.66 | 0.78 |
|  | **AUC** | 0.79 (0.69-0.89)* | 0.87 (0.79-0.95)* |
|  | **Sensitivity** | 0.48 | 0.75 |
|  | **Specificity** | 0.84 | 0.81 |
| **XGB** | **Balanced Accuracy** | 0.69 | 0.7 |
|  | **AUC** | 0.76 (0.65-0.87)* | 0.79 (0.68-0.89)* |
|  | **Sensitivity** | 0.68 | 0.68 |
|  | **Specificity** | 0.69 | 0.73 |
| **NN** | **Balanced Accuracy** | 0.57 | 0.64 |
|  | **AUC** | 0.6 (0.47-0.73) | 0.7 (0.58-0.82)* |
|  | **Sensitivity** | 0.50 | 0.63 |
|  | **Specificity** | 0.65 | 0.65 |

**Table S8.** Multi-site classification results for combined samples using different classifiers and techniques to deal with covariates (age, sex and site ID). Reported metrics are averages across CV folds. Asterisks indicate AUC scores significantly different from chance (p<0.05 Bonferroni corrected (9 classifiers)) and 95% CI are shown in brackets.

| **CV scheme** | | Intra-site CV (fixed fold-size) | Intra-site CV (fixed fold-size) | Intra-site CV (fixed fold-size) | Intra-site CV (fixed fold-size) |
| --- | --- | --- | --- | --- | --- |
| **Classification** | | Diagnosis | Diagnosis | Diagnosis | Diagnosis |
| **Age_Group** | | combined | combined | combined | combined |
| **N** | | 3857 | 3857 | 3857 | 3857 |
| **Covariates used** | | Only covariates | With covariates | No covariates | Linear regressed |
| **SVM** | **Balanced Accuracy** | 0.54 | 0.57 | 0.56 | 0.55 |
|  | **AUC** | 0.58 (0.53-0.64)* | 0.59 (0.53-0.65)* | 0.58 (0.52-0.63)* | 0.56 (0.51-0.62) |
|  | **Sensitivity** | 0.58 | 0.56 | 0.58 | 0.53 |
|  | **Specificity** | 0.50 | 0.58 | 0.53 | 0.57 |
| **PCA + SVM** | **Balanced Accuracy** | 0.53 | 0.56 | 0.55 | 0.54 |
|  | **AUC** | 0.58 (0.53-0.64)* | 0.58 (0.52-0.64) | 0.58 (0.52-0.63)* | 0.56 (0.5-0.62) |
|  | **Sensitivity** | 0.69 | 0.57 | 0.58 | 0.53 |
|  | **Specificity** | 0.38 | 0.54 | 0.53 | 0.56 |
| **RBF-SVM** | **Balanced Accuracy** | 0.51 | 0.56 | 0.56 | 0.54 |
|  | **AUC** | 0.44 (0.38-0.5) | 0.6 (0.55-0.66)* | 0.59 (0.53-0.65)* | 0.56 (0.5-0.62) |
|  | **Sensitivity** | 0.62 | 0.65 | 0.61 | 0.54 |
|  | **Specificity** | 0.41 | 0.46 | 0.51 | 0.53 |
| **PCA + RBF-SVM** | **Balanced Accuracy** | 0.53 | 0.55 | 0.55 | 0.53 |
|  | **AUC** | 0.53 (0.47-0.59) | 0.59 (0.53-0.65)* | 0.59 (0.53-0.64)* | 0.56 (0.5-0.62) |
|  | **Sensitivity** | 0.39 | 0.78 | 0.75 | 0.52 |
|  | **Specificity** | 0.67 | 0.32 | 0.36 | 0.55 |
| **LOG** | **Balanced Accuracy** | 0.56 | 0.57 | 0.56 | 0.54 |
|  | **AUC** | 0.6 (0.54-0.65)* | 0.6 (0.54-0.66)* | 0.58 (0.52-0.64)* | 0.56 (0.5-0.62) |
|  | **Sensitivity** | 0.57 | 0.58 | 0.55 | 0.53 |
|  | **Specificity** | 0.55 | 0.56 | 0.57 | 0.55 |
| **PCA + LOG** | **Balanced Accuracy** | 0.54 | 0.56 | 0.56 | 0.54 |
|  | **AUC** | 0.58 (0.53-0.64)* | 0.58 (0.52-0.64) | 0.58 (0.52-0.64)* | 0.56 (0.5-0.61) |
|  | **Sensitivity** | 0.56 | 0.56 | 0.56 | 0.53 |
|  | **Specificity** | 0.53 | 0.56 | 0.56 | 0.54 |
| **XGB** | **Balanced Accuracy** | 0.58 | 0.57 | 0.57 | 0.53 |
|  | **AUC** | 0.62 (0.57-0.68)* | 0.6 (0.54-0.65)* | 0.59 (0.53-0.64)* | 0.54 (0.48-0.6) |
|  | **Sensitivity** | 0.68 | 0.62 | 0.62 | 0.59 |
|  | **Specificity** | 0.49 | 0.52 | 0.52 | 0.46 |
| **BRFC** | **Balanced Accuracy** | 0.54 | 0.59 | 0.57 | 0.56 |
|  | **AUC** | 0.58 (0.52-0.64)* | 0.62 (0.56-0.67)* | 0.61 (0.55-0.66)* | 0.58 (0.52-0.64)* |
|  | **Sensitivity** | 0.51 | 0.57 | 0.55 | 0.57 |
|  | **Specificity** | 0.58 | 0.60 | 0.60 | 0.55 |
| **GPC** | **Balanced Accuracy** | 0.55 | 0.57 | 0.56 | 0.53 |
|  | **AUC** | 0.59 (0.53-0.65)* | 0.59 (0.53-0.64)* | 0.58 (0.52-0.64)* | 0.56 (0.5-0.61) |
|  | **Sensitivity** | 0.67 | 0.57 | 0.55 | 0.53 |
|  | **Specificity** | 0.44 | 0.56 | 0.56 | 0.53 |

**Table S9a.** Classifications performed for medicated vs unmedicated OCD patients using different classifiers and techniques to deal with covariates (age, sex and site ID). Reported metrics are averages across CV folds. Asterisks indicate AUC scores significantly different from chance (p<0.05 Bonferroni corrected (9 classifiers)) and 95% CI are shown in brackets.

| **CV scheme** | | Intra-site CV (fixed fold-size) | Intra-site CV (fixed fold-size) | Intra-site CV (fixed fold-size) | Intra-site CV (fixed fold-size) |
| --- | --- | --- | --- | --- | --- |
| **Classification** | | Medication | Medication | Medication | Medication |
| **Age_Group** | | combined | combined | combined | combined |
| **N** | | 1745 | 1745 | 1745 | 1745 |
| **Covariates used** | | Only covariates | With covariates | No covariates | Linear regressed |
| **SVM** | **Balanced Accuracy** | 0.77 | 0.76 | 0.67 | 0.50 |
|  | **AUC** | 0.84 (0.78-0.9)* | 0.83 (0.77-0.89)* | 0.73 (0.65-0.8)* | 0.49 (0.4-0.57) |
|  | **Sensitivity** | 0.76 | 0.77 | 0.66 | 0.30 |
|  | **Specificity** | 0.77 | 0.76 | 0.68 | 0.70 |
| **PCA + SVM** | **Balanced Accuracy** | 0.76 | 0.67 | 0.67 | 0.51 |
|  | **AUC** | 0.8 (0.74-0.87)* | 0.73 (0.66-0.8)* | 0.72 (0.65-0.8)* | 0.48 (0.4-0.57) |
|  | **Sensitivity** | 0.67 | 0.65 | 0.65 | 0.44 |
|  | **Specificity** | 0.85 | 0.69 | 0.68 | 0.58 |
| **RBF-SVM** | **Balanced Accuracy** | 0.77 | 0.72 | 0.69 | 0.50 |
|  | **AUC** | 0.85 (0.79-0.91)* | 0.78 (0.71-0.85)* | 0.75 (0.68-0.82)* | 0.49 (0.4-0.57) |
|  | **Sensitivity** | 0.76 | 0.72 | 0.69 | 0.11 |
|  | **Specificity** | 0.78 | 0.73 | 0.69 | 0.90 |
| **PCA + RBF-SVM** | **Balanced Accuracy** | 0.76 | 0.69 | 0.68 | 0.52 |
|  | **AUC** | 0.84 (0.78-0.9)* | 0.75 (0.68-0.82)* | 0.75 (0.67-0.82)* | 0.51 (0.43-0.6) |
|  | **Sensitivity** | 0.71 | 0.68 | 0.67 | 0.36 |
|  | **Specificity** | 0.82 | 0.69 | 0.69 | 0.68 |
| **LOG** | **Balanced Accuracy** | 0.77 | 0.77 | 0.67 | 0.51 |
|  | **AUC** | 0.88 (0.82-0.93)* | 0.85 (0.8-0.91)* | 0.73 (0.66-0.81)* | 0.5 (0.41-0.59) |
|  | **Sensitivity** | 0.77 | 0.77 | 0.65 | 0.29 |
|  | **Specificity** | 0.78 | 0.76 | 0.68 | 0.72 |
| **PCA + LOG** | **Balanced Accuracy** | 0.77 | 0.68 | 0.67 | 0.51 |
|  | **AUC** | 0.86 (0.8-0.91)* | 0.73 (0.66-0.81)* | 0.72 (0.65-0.8)* | 0.51 (0.43-0.6) |
|  | **Sensitivity** | 0.69 | 0.66 | 0.65 | 0.35 |
|  | **Specificity** | 0.85 | 0.70 | 0.68 | 0.67 |
| **XGB** | **Balanced Accuracy** | 0.78 | 0.76 | 0.67 | 0.55 |
|  | **AUC** | 0.87 (0.82-0.92)* | 0.86 (0.8-0.91)* | 0.74 (0.66-0.81)* | 0.57 (0.49-0.66) |
|  | **Sensitivity** | 0.74 | 0.73 | 0.61 | 0.46 |
|  | **Specificity** | 0.82 | 0.80 | 0.72 | 0.63 |
| **BRFC** | **Balanced Accuracy** | 0.73 | 0.71 | 0.67 | 0.55 |
|  | **AUC** | 0.84 (0.78-0.9)* | 0.78 (0.72-0.85)* | 0.74 (0.67-0.81)* | 0.59 (0.5-0.67) |
|  | **Sensitivity** | 0.74 | 0.68 | 0.62 | 0.53 |
|  | **Specificity** | 0.72 | 0.75 | 0.72 | 0.57 |
| **GPC** | **Balanced Accuracy** | 0.77 | 0.70 | 0.67 | 0.52 |
|  | **AUC** | 0.87 (0.82-0.92)* | 0.77 (0.7-0.84)* | 0.73 (0.66-0.81)* | 0.52 (0.44-0.61) |
|  | **Sensitivity** | 0.77 | 0.65 | 0.64 | 0.50 |
|  | **Specificity** | 0.77 | 0.75 | 0.70 | 0.54 |

**Table S9b.** Classifications performed for healthy controls vs unmedicated OCD patients using different classifiers and techniques to deal with covariates (age, sex and site ID). Reported metrics are averages across CV folds. Asterisks indicate AUC scores significantly different from chance (p<0.05 Bonferroni corrected (9 classifiers)) and 95% CI are shown in brackets.

| **CV scheme** | | Intra-site CV (fixed fold-size) | Intra-site CV (fixed fold-size) | Intra-site CV (fixed fold-size) | Intra-site CV (fixed fold-size) |
| --- | --- | --- | --- | --- | --- |
| **Classification** | | Diagnosis | Diagnosis | Diagnosis | Diagnosis |
| **Age_Group** | | combined | combined | combined | combined |
| **N** | | 2797 | 2797 | 2797 | 2797 |
| **Covariates used** | | Only covariates | With covariates | No covariates | Linear regressed |
| **SVM** | **Balanced Accuracy** | 0.63 | 0.62 | 0.54 | 0.51 |
|  | **AUC** | 0.65 (0.59-0.72)* | 0.65 (0.59-0.72)* | 0.57 (0.5-0.64) | 0.49 (0.42-0.56) |
|  | **Sensitivity** | 0.74 | 0.67 | 0.56 | 0.48 |
|  | **Specificity** | 0.52 | 0.57 | 0.51 | 0.54 |
| **PCA + SVM** | **Balanced Accuracy** | 0.62 | 0.54 | 0.54 | 0.51 |
|  | **AUC** | 0.65 (0.59-0.72)* | 0.56 (0.49-0.63) | 0.56 (0.49-0.63) | 0.51 (0.44-0.58) |
|  | **Sensitivity** | 0.82 | 0.59 | 0.58 | 0.49 |
|  | **Specificity** | 0.43 | 0.50 | 0.50 | 0.53 |
| **RBF-SVM** | **Balanced Accuracy** | 0.64 | 0.54 | 0.51 | 0.50 |
|  | **AUC** | 0.68 (0.62-0.74)* | 0.59 (0.52-0.66) | 0.58 (0.51-0.65) | 0.53 (0.46-0.6) |
|  | **Sensitivity** | 0.71 | 0.32 | 0.05 | 0.21 |
|  | **Specificity** | 0.57 | 0.76 | 0.96 | 0.80 |
| **PCA + RBF-SVM** | **Balanced Accuracy** | 0.64 | 0.50 | 0.51 | 0.50 |
|  | **AUC** | 0.67 (0.61-0.73)* | 0.61 (0.54-0.68)* | 0.61 (0.55-0.68)* | 0.51 (0.44-0.58) |
|  | **Sensitivity** | 0.74 | 0.06 | 0.01 | 0.01 |
|  | **Specificity** | 0.54 | 0.95 | 1.00 | 1.00 |
| **LOG** | **Balanced Accuracy** | 0.64 | 0.61 | 0.56 | 0.52 |
|  | **AUC** | 0.68 (0.61-0.74)* | 0.66 (0.59-0.72)* | 0.58 (0.51-0.65) | 0.53 (0.46-0.6) |
|  | **Sensitivity** | 0.71 | 0.64 | 0.57 | 0.51 |
|  | **Specificity** | 0.56 | 0.59 | 0.55 | 0.52 |
| **PCA + LOG** | **Balanced Accuracy** | 0.64 | 0.54 | 0.54 | 0.51 |
|  | **AUC** | 0.66 (0.6-0.73)* | 0.57 (0.5-0.64) | 0.57 (0.5-0.63) | 0.52 (0.45-0.59) |
|  | **Sensitivity** | 0.72 | 0.56 | 0.56 | 0.36 |
|  | **Specificity** | 0.56 | 0.53 | 0.53 | 0.65 |
| **XGB** | **Balanced Accuracy** | 0.58 | 0.54 | 0.54 | 0.51 |
|  | **AUC** | 0.67 (0.61-0.73)* | 0.61 (0.55-0.68)* | 0.57 (0.5-0.64) | 0.53 (0.46-0.6) |
|  | **Sensitivity** | 0.30 | 0.21 | 0.22 | 0.18 |
|  | **Specificity** | 0.86 | 0.86 | 0.85 | 0.84 |
| **BRFC** | **Balanced Accuracy** | 0.61 | 0.56 | 0.55 | 0.52 |
|  | **AUC** | 0.64 (0.58-0.71)* | 0.6 (0.54-0.67) | 0.58 (0.51-0.65) | 0.52 (0.45-0.59) |
|  | **Sensitivity** | 0.67 | 0.52 | 0.49 | 0.40 |
|  | **Specificity** | 0.54 | 0.60 | 0.61 | 0.64 |
| **GPC** | **Balanced Accuracy** | 0.55 | 0.55 | 0.55 | 0.52 |
|  | **AUC** | 0.66 (0.6-0.72)* | 0.59 (0.52-0.66) | 0.57 (0.5-0.64) | 0.52 (0.45-0.59) |
|  | **Sensitivity** | 0.30 | 0.31 | 0.46 | 0.51 |
|  | **Specificity** | 0.81 | 0.79 | 0.64 | 0.52 |

**Table S9c.** Classifications performed for healthy controls vs medicated OCD patients using different classifiers and techniques to deal with covariates (age, sex and site ID)**.** Reported metrics are averages across CV folds. Asterisks indicate AUC scores significantly different from chance (p<0.05 Bonferroni corrected (9 classifiers)) and 95% CI are shown in brackets.

| **CV scheme** | | Intra-site CV (fixed fold-size) | Intra-site CV (fixed fold-size) | Intra-site CV (fixed fold-size) | Intra-site CV (fixed fold-size) |
| --- | --- | --- | --- | --- | --- |
| **Classification** | | Diagnosis | Diagnosis | Diagnosis | Diagnosis |
| **Age_Group** | | combined | combined | combined | combined |
| **N** | | 2692 | 2692 | 2692 | 2692 |
| **Covariates used** | | Only covariates | With covariates | No covariates | Linear regressed |
| **SVM** | **Balanced Accuracy** | 0.64 | 0.64 | 0.62 | 0.57 |
|  | **AUC** | 0.71 (0.65-0.77)* | 0.71 (0.65-0.78)* | 0.65 (0.59-0.72)* | 0.6 (0.53-0.67)* |
|  | **Sensitivity** | 0.63 | 0.64 | 0.60 | 0.56 |
|  | **Specificity** | 0.65 | 0.65 | 0.63 | 0.58 |
| **PCA + SVM** | **Balanced Accuracy** | 0.64 | 0.62 | 0.61 | 0.58 |
|  | **AUC** | 0.68 (0.62-0.75)* | 0.66 (0.6-0.73)* | 0.65 (0.59-0.72)* | 0.6 (0.53-0.67)* |
|  | **Sensitivity** | 0.55 | 0.61 | 0.60 | 0.56 |
|  | **Specificity** | 0.72 | 0.63 | 0.62 | 0.59 |
| **RBF-SVM** | **Balanced Accuracy** | 0.67 | 0.66 | 0.64 | 0.58 |
|  | **AUC** | 0.73 (0.67-0.79)* | 0.72 (0.66-0.78)* | 0.7 (0.64-0.76)* | 0.6 (0.54-0.67)* |
|  | **Sensitivity** | 0.70 | 0.63 | 0.61 | 0.52 |
|  | **Specificity** | 0.64 | 0.69 | 0.68 | 0.63 |
| **PCA + RBF-SVM** | **Balanced Accuracy** | 0.66 | 0.64 | 0.63 | 0.56 |
|  | **AUC** | 0.72 (0.66-0.78)* | 0.69 (0.63-0.76)* | 0.69 (0.62-0.75)* | 0.6 (0.53-0.67)* |
|  | **Sensitivity** | 0.69 | 0.61 | 0.60 | 0.50 |
|  | **Specificity** | 0.62 | 0.68 | 0.67 | 0.63 |
| **LOG** | **Balanced Accuracy** | 0.65 | 0.66 | 0.62 | 0.57 |
|  | **AUC** | 0.73 (0.67-0.79)* | 0.73 (0.67-0.79)* | 0.66 (0.6-0.73)* | 0.6 (0.53-0.67)* |
|  | **Sensitivity** | 0.67 | 0.66 | 0.61 | 0.57 |
|  | **Specificity** | 0.62 | 0.65 | 0.64 | 0.57 |
| **PCA + LOG** | **Balanced Accuracy** | 0.64 | 0.62 | 0.62 | 0.56 |
|  | **AUC** | 0.71 (0.65-0.77)* | 0.66 (0.6-0.73)* | 0.66 (0.59-0.72)* | 0.59 (0.52-0.66)* |
|  | **Sensitivity** | 0.64 | 0.61 | 0.62 | 0.57 |
|  | **Specificity** | 0.65 | 0.63 | 0.62 | 0.56 |
| **XGB** | **Balanced Accuracy** | 0.61 | 0.62 | 0.58 | 0.55 |
|  | **AUC** | 0.74 (0.68-0.8)* | 0.73 (0.67-0.79)* | 0.66 (0.6-0.73)* | 0.62 (0.55-0.69)* |
|  | **Sensitivity** | 0.32 | 0.35 | 0.29 | 0.19 |
|  | **Specificity** | 0.91 | 0.89 | 0.87 | 0.90 |
| **BRFC** | **Balanced Accuracy** | 0.66 | 0.63 | 0.61 | 0.57 |
|  | **AUC** | 0.71 (0.65-0.78)* | 0.69 (0.63-0.75)* | 0.66 (0.6-0.73)* | 0.59 (0.52-0.66) |
|  | **Sensitivity** | 0.73 | 0.53 | 0.50 | 0.46 |
|  | **Specificity** | 0.59 | 0.73 | 0.72 | 0.69 |
| **GPC** | **Balanced Accuracy** | 0.62 | 0.61 | 0.61 | 0.57 |
|  | **AUC** | 0.72 (0.65-0.78)* | 0.69 (0.63-0.75)* | 0.66 (0.59-0.73)* | 0.6 (0.53-0.67)* |
|  | **Sensitivity** | 0.38 | 0.40 | 0.53 | 0.57 |
|  | **Specificity** | 0.86 | 0.82 | 0.69 | 0.56 |

**Table S10a**. Features important for Medicated vs Unmedicated OCD classification in combined samples using covariate regressed FreeSurfer data. Features listed in this table survived FDR correction in >50% CV folds.

| **Feature** | **Consistency (%)** |
| --- | --- |
| L_insula_thickavg | 100.0 |
| L_parsorbitalis_thickavg | 100.0 |
| R_entorhinal_surfavg | 100.0 |
| R_temporalpole_surfavg | 100.0 |
| L_bankssts_thickavg | 100.0 |
| L_inferiortemporal_thickavg | 100.0 |
| LLatVent | 100.0 |
| R_transversetemporal_thickavg | 90.0 |
| Lthal | 90.0 |
| RLatVent | 90.0 |
| L_caudalanteriorcingulate_thickavg | 80.0 |
| Lpal | 80.0 |
| L_temporalpole_surfavg | 80.0 |
| R_superiortemporal_thickavg | 80.0 |
| R_frontalpole_thickavg | 80.0 |
| L_superiortemporal_thickavg | 70.0 |
| R_posteriorcingulate_thickavg | 70.0 |
| R_supramarginal_thickavg | 70.0 |
| L_paracentral_surfavg | 70.0 |
| L_isthmuscingulate_thickavg | 70.0 |
| R_caudalanteriorcingulate_thickavg | 70.0 |
| L_medialorbitofrontal_thickavg | 60.0 |
| R_caudalmiddlefrontal_thickavg | 60.0 |
| L_transversetemporal_thickavg | 60.0 |

**Table S10b.** Features important for Medicated vs Unmedicated OCD classification in adult samples using covariate regressed FreeSurfer data. Features listed in this table survived FDR correction in >50% CV folds.

| **Feature** | **Consistency (%)** |
| --- | --- |
| R_transversetemporal_thickavg | 100.0 |
| L_superiortemporal_thickavg | 100.0 |
| Lthal | 100.0 |
| RLatVent | 100.0 |
| L_bankssts_thickavg | 100.0 |
| L_caudalanteriorcingulate_thickavg | 100.0 |
| L_insula_thickavg | 100.0 |
| L_isthmuscingulate_thickavg | 100.0 |
| L_medialorbitofrontal_thickavg | 100.0 |
| L_parsorbitalis_thickavg | 100.0 |
| L_inferiortemporal_thickavg | 100.0 |
| R_caudalanteriorcingulate_thickavg | 100.0 |
| R_supramarginal_thickavg | 90.0 |
| Rthal | 90.0 |
| R_middletemporal_thickavg | 90.0 |
| LLatVent | 90.0 |
| R_posteriorcingulate_thickavg | 90.0 |
| R_superiortemporal_thickavg | 90.0 |
| L_paracentral_surfavg | 90.0 |
| Lpal | 90.0 |
| L_caudalmiddlefrontal_thickavg | 80.0 |
| R_temporalpole_surfavg | 80.0 |
| R_caudalmiddlefrontal_thickavg | 80.0 |
| R_frontalpole_thickavg | 80.0 |
| L_fusiform_thickavg | 80.0 |
| R_precuneus_thickavg | 80.0 |
| subcort_ICV | 70.0 |
| L_temporalpole_surfavg | 70.0 |
| L_entorhinal_surfavg | 60.0 |
| R_precentral_thickavg | 60.0 |
| R_paracentral_thickavg | 60.0 |
| Raccumb | 60.0 |
| L_bankssts_surfavg | 60.0 |
| L_transversetemporal_thickavg | 60.0 |

**Table S11.** Features important for Early vs Late OCD classification in combined samples using covariate regressed FreeSurfer data. Features listed in this table survived FDR correction in >50% CV folds.

| **Feature** | **Consistency (%)** |
| --- | --- |
| Laccumb | 90.0 |
| Lput | 90.0 |
| L_frontalpole_thickavg | 90.0 |
| R_postcentral_thickavg | 90.0 |
| R_caudalmiddlefrontal_thickavg | 80.0 |
| R_superiorfrontal_thickavg | 80.0 |
| subcort_ICV | 70.0 |
| Lpal | 70.0 |
| L_lingual_surfavg | 70.0 |
| R_lateraloccipital_surfavg | 70.0 |
| L_parstriangularis_thickavg | 70.0 |
| L_caudalanteriorcingulate_thickavg | 60.0 |

Online-Only References

1. Rasmussen, C. E., Williams, C. K. I. *Gaussian processes for machine learning.* *International journal of neural systems* vol. 14 (2004).

2. Probst, P., Wright, M. N. & Boulesteix, A. L. Hyperparameters and tuning strategies for random forest. *Wiley Interdiscip. Rev. Data Min. Knowl. Discov.* 1–19 (2019) doi:10.1002/widm.1301.

3. Boulesteix, A. L., Janitza, S., Kruppa, J. & König, I. R. Overview of random forest methodology and practical guidance with emphasis on computational biology and bioinformatics. *Wiley Interdiscip. Rev. Data Min. Knowl. Discov.* **2**, 493–507 (2012).

4. Strobl, C., Boulesteix, A. L., Zeileis, A. & Hothorn, T. Bias in random forest variable importance measures: Illustrations, sources and a solution. *BMC Bioinformatics* **8**, (2007).

5. Chen, C., Liaw, A. & Breiman, L. Using random forest to learn imbalanced data. *Unpubl. Tech. Rep.* 1–12 (2004) doi:ley.edu/sites/default/files/tech-reports/666.pdf.

6. Hastie, T., Tibshirani, R. & Friedman, J. *The Elements of Statistical Learning (2nd edition)*. *Elements* (2009). doi:10.1007/978-0-387-84858-7.

7. Nair, V. & Hinton, G. E. Rectified Linear Units Improve Restricted Boltzmann Machines. *Proc. 27th Int. Conf. Mach. Learn.* (2010) doi:10.1.1.165.6419.

8. Kingma, D. P. & Ba, J. Adam: A Method for Stochastic Optimization. 1–15 (2014).

9. Varoquaux, G. Cross-validation failure: Small sample sizes lead to large error bars. *Neuroimage* 1–10 (2017) doi:10.1016/j.neuroimage.2017.06.061.

10. Combrisson, E. & Jerbi, K. Exceeding chance level by chance: The caveat of theoretical chance levels in brain signal classification and statistical assessment of decoding accuracy. *J. Neurosci. Methods* **250**, 126–136 (2015).

11. Pedregosa, F. *et al.* Scikit-learn: Machine Learning in Python. *J. Mach. Learn. Res.* **12**, 2825–2830 (2012).

12. Mason, S. J. & Graham, N. E. Areas beneath the relative operating characteristics (ROC) and relative operating levels (ROL) curves. *Q. J. R. Meteorol. Soc.* **128**, 2145–2166 (2002).

13. Hanley, J. A. & McNeil, B. J. The meaning and use of the area under a receiver operating characteristic (ROC) curve. *Radiology* **143**, 29–36 (1982).

14. Bradley, A. P. The use of the area under the ROC curve in the evaluation of machine learning algorithms. *Pattern Recognit.* **30**, 1145–1159 (1997).

15. Bengio, Y. & Grandvalet, Y. No Unbiased Estimator of the Variance of K-Fold Cross-Validation. *J. Mach. Learn. Res.* **5**, 1089–1105 (2004).

16. Breiman, L. E. O. Random Forests. *Mach. Learn.* **45**, 5–32 (2001).

17. Hapfelmeier, A. & Ulm, K. A new variable selection approach using Random Forests. *Comput. Stat. Data Anal.* **60**, 50–69 (2013).

18. Strobl, C. An Introduction to Recursive Partitioning: Rationale, Application and Characteristics of Classification. *Psychol Methods* **14**, 323–348 (2010).

19. Winkler, A. M. *et al.* Non-parametric combination and related permutation tests for neuroimaging. *Hum. Brain Mapp.* (2016) doi:10.1002/hbm.23115.

20. Boedhoe, P. S. W. *et al.* Cortical Abnormalities Associated With Pediatric and Adult Obsessive-Compulsive Disorder: Findings From the ENIGMA Obsessive-Compulsive Disorder Working Group. *Am. J. Psychiatry* **175**, 453–462 (2018).

21. Boedhoe, P. S. W. *et al.* Distinct Subcortical Volume Alterations in Pediatric and Adult OCD: A Worldwide Meta- and Mega-Analysis. *Am. J. Psychiatry* **174**, 60–69 (2017).
